# Characterization of winged helix domain fusion endonucleases as N6-methyladenine-dependent type IV restriction systems

**DOI:** 10.3389/fmicb.2024.1286822

**Published:** 2024-04-09

**Authors:** Igor Helbrecht, Daniel Heiter, Weiwei Yang, Tamas Vincze, Andrew Hanneman, Thomas Lutz, Laurence Ettwiller, Matthias Bochtler, Shuang-yong Xu

**Affiliations:** ^1^New England Biolabs, Inc., Ipswich, MA, United States; ^2^Institute of Biochemistry and Biophysics, Polish Academy of Sciences, Warsaw, Poland; ^3^International Institute of Molecular and Cell Biology, Warsaw, Poland

**Keywords:** winged helix fusion endonucleases, HNH endonuclease, GIY-YIG endonuclease, PLD family endonuclease, N6mA-dependent restriction system, genome conflict

## Abstract

Winged helix (wH) domains, also termed winged helix-turn-helix (wHTH) domains, are widespread in all kingdoms of life and have diverse roles. In the context of DNA binding and DNA modification sensing, some eukaryotic wH domains are known as sensors of non-methylated CpG. In contrast, the prokaryotic wH domains in DpnI and HhiV4I act as sensors of adenine methylation in the 6mApT (N6-methyladenine, 6mA, or N6mA) context. DNA-binding modes and interactions with the probed dinucleotide are vastly different in the two cases. Here, we show that the role of the wH domain as a sensor of adenine methylation is widespread in prokaryotes. We present previously uncharacterized examples of PD-(D/E)XK—wH (FcyTI, Psp4BI), PUA—wH—HNH (HtuIII), wH—GIY-YIG (Ahi29725I, Apa233I), and PLD—wH (Aba4572I, CbaI) fusion endonucleases that sense adenine methylation in the Dam^+^ Gm6ATC sequence contexts. Representatives of the wH domain endonuclease fusion families with the exception of the PLD—wH family could be purified, and an *in vitro* preference for adenine methylation in the Dam context could be demonstrated. Like most other modification-dependent restriction endonucleases (MDREs, also called type IV restriction systems), the new fusion endonucleases except those in the PD-(D/E)XK—wH family cleave close to but outside the recognition sequence. Taken together, our data illustrate the widespread combinatorial use of prokaryotic wH domains as adenine methylation readers. Other potential 6mA sensors in modified DNA are also discussed.

## Introduction

In most restriction–modification (R-M) scenarios, nucleobase modification serves as a mark of self and provides protection against endonuclease digestion. In some cases, however, phages have learned to exploit this principle by modifying their own DNA, either by incorporation of non-standard nucleoside triphosphates or by post-replicative modifications catalyzed either by host or phage enzymes. Modification-dependent restriction endonucleases (MDREs) specifically target such modified DNA (modified base or backbone). The MDREs come in two main groups, distinguished by the presence or absence of nucleoside triphosphate (NTP)-consuming motor proteins. The NTP-independent proteins are typically modular, with separate modification sensing and DNA cleavage domains. Because of this architecture, DNA cleavage typically takes place at a distance from the site of modification. For some enzymes, a single site is sufficient, but typically, cleavage is most efficient when it is directed by appropriately spaced modifications, which cooperate to position an endonuclease dimer for a double strand (ds) cut in the DNA.

The catalytic domains present in restriction can be grouped into the almost universally used hydrolases (Orlowski and Bujnicki, 2008) and the very rarely used lyases (Miyazono et al., 2014). The hydrolases, in turn, can be grouped into a surprisingly small set of phylogenetically unrelated enzyme groups. PD-(D/E)XK enzymes are named for characteristic amino acids (aa) built around a central β-sheet, which harbors one or two catalytic Mg^2+^ ions (Pingoud et al., 2005). The metal ions are held in place in part by the D and D or E (abbreviated as D/E) interacting residues, which, together with a K residue, activate a water molecule for direct inline attack on the scissile phosphate (Bujnicki and Rychlewski, 2001; Kosinski et al., 2005). HNH enzymes, also called ββα-Me enzymes or His-Me finger enzymes (Jablonska et al., 2017; Wu et al., 2020), harbor a single metal cation in their active site. Metal identity requirements are less strict than for PD-(D/E)XK enzymes. Many divalent transition metal ions are acceptable (Pommer et al., 2001). Like PD-(D/E)XK enzymes, the HNH enzymes are believed to catalyze attack on the scissile phosphate by a water molecule. However, water activation is not by a lysine residue but by the first histidine of the HNH motif (Flick et al., 1998; Sokolowska et al., 2009). GIY-YIG enzymes (Dunin-Horkawicz et al., 2006; Kaminska et al., 2008) also bind a single metal cation in the active site. These enzymes activate the water molecule with a tyrosine residue, most likely from the GIY motif (Sokolowska et al., 2011). Finally, there are also completely metal-independent endonuclease domains. They resemble phospholipase D; therefore, the enzymes containing them are known as PLD endonucleases (Grazulis et al., 2005; Chan et al., 2007). The PLD enzymes are believed to catalyze phosphodiester cleavage via a covalent intermediate (Sasnauskas et al., 2010).

The modification sensor domains, like the endonuclease domains, are now understood to be classifiable into only a few groups of phylogenetically unrelated sensors. The largest group of sensors is the PUA (PseudoUridine synthase and Archaeosine transglycosylase) superfamily (Lutz et al., 2019). PUA superfamily sensors comprise SRA (SET and Ring finger Associated) domains with specificity for 5mC, as in MspJI (Cohen-Karni et al., 2011), and related domains (Kazrani et al., 2014; Shao et al., 2014), originally also termed SRA domains, with specificity for 5-hydroxymethylcytosine (5hmC) and glucosyl-5-hydroxymethyl-cytosine (g5hmC), as in the PvuRts1I family of restriction endonucleases (Janosi et al., 1994; Borgaro and Zhu, 2013). The PUA superfamily also comprises EVE (according to the PDB identifier 2eve for a prototypical protein; Bertonati et al., 2009) domains specific for 5mC and 5hmC, as found in VcaM4I (Pastor et al., 2021), and YTH (YT521-B Homologs) domains (YTH-McrB/NTPase fusion) specific for 6-methyladenine (6mA) (Iyer et al., 2006; Hosford et al., 2020; Xu et al., 2020), as well as ASCH (ASC-1 Homology) domains. Bioinformatic analysis has suggested that ASCH domains might be 6mA readers (Iyer et al., 2016), but this prediction has not been confirmed by experimental data so far. Instead, it has been shown that the *E. coli* YqfB, an ASCH domain protein, is able to hydrolyze various N4-acylated cytosines (4acC) and cytidines (Stanislauskiene et al., 2020). All PUA superfamily domains are engaged in nucleotide flipping (Cerrudo et al., 2014; Pastor et al., 2021). Irrespective of their detailed specificity, they scrutinize the modified base in a dedicated pocket of the enzyme (Roberts and Cheng, 1998; Horton et al., 2014). It has been shown that *E. coli* McrBC endonuclease also recognizes modified cytosines by base flipping (Sukackaite et al., 2012).

Apart from the PUA superfamily, other modification sensor domains may also be involved in restriction, such as the NEco domain in EcoKMcrA with affinity for 5mC and 5hmC (Czapinska et al., 2018). Unlike the PUA superfamily domains, the NEco domain senses 5mC or 5hmC without nucleotide flipping in the context of dsDNA (Slyvka et al., 2019). Finally, a winged helix (wH) domain has been described as a 6mA sensor in DpnI. Like the NEco domain, the wH (winged helix) domain senses nucleobase modifications in the context of dsDNA without flipping (Mierzejewska et al., 2014). However, in contrast to the NEco domain, it has specificity for 6mA rather than 5mC. Also, in contrast to NEco, which recognizes methyl groups of fully methylated CpG in two separate pockets, the wH domain recognizes methyl groups of fully methylated ApT in a single pocket, exploiting their proximity in space. The wH domain in DpnI is unusual in being fused to a nuclease domain, which has a separate sequence (GATC) and modification (6mA) specificity (Siwek et al., 2012). Therefore, it acts more like an effector domain in type IIE enzymes (Senesac and Allen, 1995; Roberts et al., 2003), except that both the nuclease and sensor/effector domain are specific for methylated rather than non-methylated DNA.

**Figure 1:**
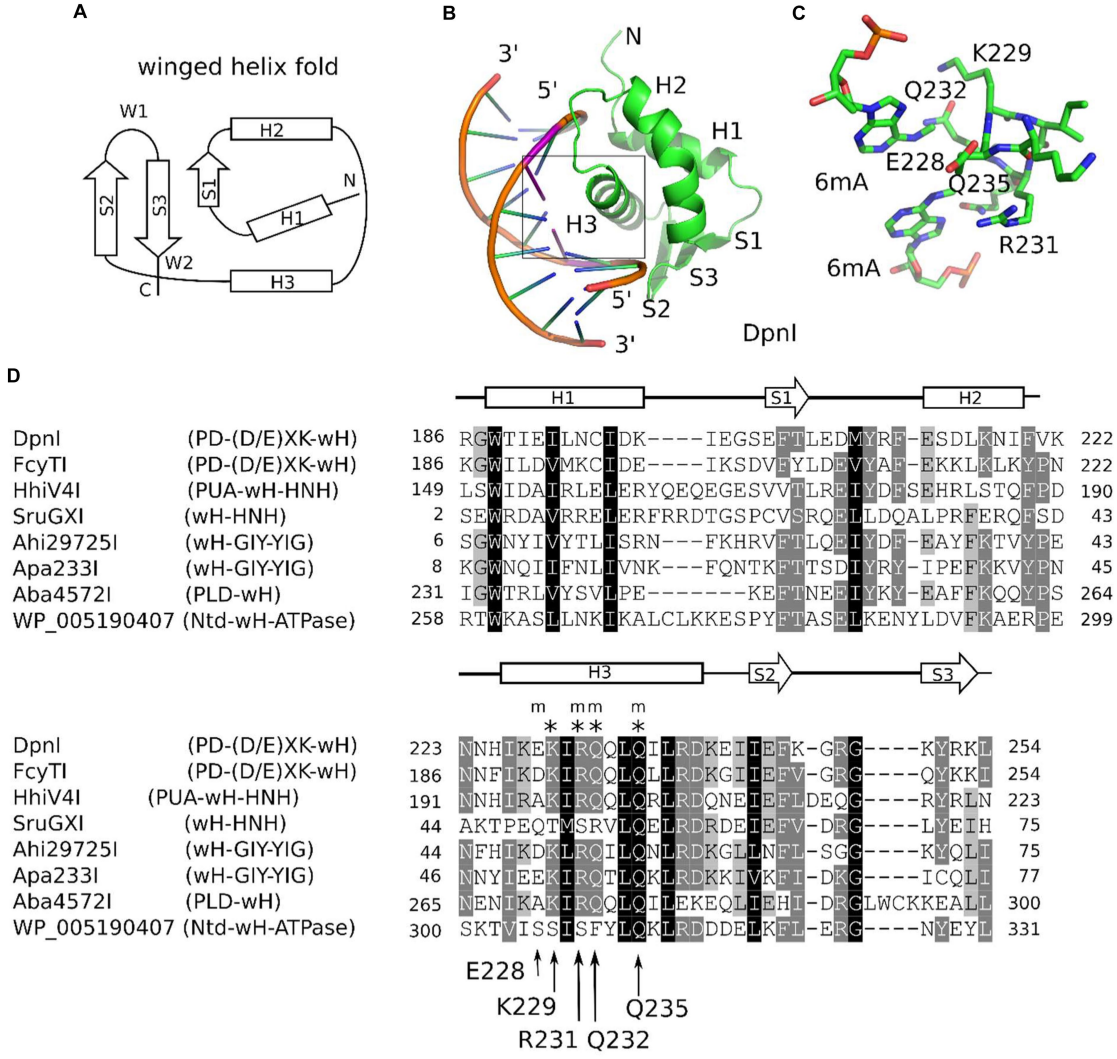
Winged helix (wH) domain fold and role as a methylation sensor. **(A)** Canonical wH fold. **(B)** Cartoon representation of the DpnI wH domain bound to fully methylated DNA, based on the crystal structure (Siwek et al., 2012). **(C)** Methyl binding region of the DpnI wH domain. **(D)** Alignment of representative winged helix domains in endonuclease or NTPase fusions. The secondary structure annotation is based on the DpnI experimental structure, analyzed for secondary structure elements using DSSP (Kabsch and Sander, 1983). DpnI residues that are involved in the formation of the pocket for the methyl groups (of DNA methylated in both strands) are marked by an “m,” and those that are involved in hydrogen bonding with the nucleobases of the GATC target sequence of DpnI are marked by an asterisk (“*”). Their identities and residue numbers in case of DpnI are indicated below the alignment (with reference to the DpnI structure with PDB accession 4kyw; Mierzejewska et al., 2014). Note the strong overlap between methyl pocket-forming residues and residues that are involved in target sequence selection.

Winged helix (wH) domains are a group of DNA-binding domains that belong to the superfamily of helix-turn-helix (HTH) proteins (Brennan, 1993; Lai et al., 1993; Gajiwala and Burley, 2000). Structurally, canonical winged helix domains consist of an N-terminal α-helices and β-strand, the HTH motif, and a β-hairpin. The “wings” of the motif are the loops connecting the strands of the β-hairpin and immediately downstream of it (Iyer et al., 2016; Figure 1A). Winged helix motifs were first found in transcription factors, but it is now clear that they also have roles in transcription initiation complexes (Teichmann et al., 2012), in the binding of left-handed Z-DNA (Schwartz et al., 2001) or RNA (Tang et al., 2021), or in protein–protein interactions (Wah et al., 1997). In transcription factors, wH domains tend to interact with DNA, just as would be expected for the HTH motif that is embedded within them. In other words, they insert the second helix of the HTH motif, which is the third helix of the wH domain, into the major groove of DNA (Gajiwala and Burley, 2000). However, other DNA-binding modes are also possible in special cases (Gajiwala et al., 2000; Wolberger and Campbell, 2000). A recent example of such alternative binding modes is the complexes of eukaryotic winged helix domains with dsDNA containing non-methylated CpG (Stielow et al., 2021; Becht et al., 2023; Weber et al., 2023). A winged helix motif in a restriction endonuclease (REase) was first noticed in the DNA-binding domain of FokI (Wah et al., 1997), but this particular wH domain does not appear to be involved in interactions with DNA.

The role of the winged helix domain in adenine methylation sensing was first noticed in DpnI. DpnI is a G6mATC-specific endonuclease that cleaves within the recognition sequence and has a strong preference for DNA that is adenine-methylated in both strands (Siwek et al., 2012). In DpnI, the winged helix domain plays the role of an effector domain that senses 6mA separately from and with slightly relaxed sequence specificity compared to the PD-(D/E)XK nuclease domain (Mierzejewska et al., 2014; Figures 1B,C). More recently, a winged helix domain was also implicated in the sensing of adenine methylation, also in the G6mATC context. HHPV4I (also called HhiV4I; Lu et al., 2023) is a three-domain enzyme, with a PUA (SRA)-like domain at the N-terminus, a winged helix domain in the middle, and an HNH endonuclease domain at the C-terminus. The PUA superfamily domain, described as an SRA domain by Lu et al., appears not to be involved in DNA modification sensing (Lu et al., 2023). By contrast, the winged helix domain directed preferential cleavage of Dam^+^ over Dam^−^ DNA, and it has much higher affinity to Dam^+^ than to Dam^−^ DNA in gel shift experiments. In contrast to DpnI, HHPV4I (HhiV4I) cleaves at a distance from the site of adenine methylation, suggesting that the endonuclease domain is directed by the winged helix domain and does not sense adenine methylation on its own (Lu et al., 2023).

In this study, we show that the winged helix domain is widely used as an adenine methylation sensor in MDREs (Figure 1D). We present additional examples of proteins that share the PD-(D/E)XK—wH architecture with DpnI or the PUA—wH—HNH architecture with HHPV4I (HhiV4I). Additionally, we show that the wH domain is can also be naturally paired with an HNH domain in the absence of a PUA superfamily domain, with a GIY-YIG endonuclease domain, with a PLD (phospholipase D) nuclease domain, or with an NTPase (GTPase/ATPase) domain. For the PD-(D/E)XK—wH, PUA—wH—HNH, wH—HNH, wH—GIY-YIG, and PLD—wH enzymes, we detect Dam^+^-dependent toxicity in *E. coli* cells, either by tight binding or digestion near the modified sites. For the fusion endonucleases PD-(D/E)XK—wH, PUA—wH—HNH, wH—HNH, and wH—GIY-YIG, but not the PLD—wH enzymes, we find representatives that are active also *in vitro*, and we show that their preferred substrate is fully methylated DNA with one enzyme exception. Unlike DpnI, many of the new wH fusion endonucleases cleave DNA outside the G6mATC recognition sequence and two modified sites in tandem with a short spacer, enhance their cleavage activity.

## Materials and methods

### Materials

*E. coli* T7 expression strains C2566 (Dam^+^) and its isogenic Dam-deficient strain ER2948 [constructed and provided by Dr. Lise Raleigh, New England Biolabs (NEB)], expression vector pTXB1, pBR322, phage λ DNA (Dam^+^ or Dam^−^), 2-log (1 kb plus) DNA ladder, chitin beads, restriction enzymes, EcoGII methylase (frequent adenine methylase), Q5 DNA polymerase PCR master mix and cloning kit (Hi-Fi DNA assembly enzyme mix), NEBExpress Ni-NTA magnetic beads, and dZTP (2-aminoadenine triphosphate is abbreviated as base Z in the literature, here we use dZ to denote the 2’-deoxynucleoside) were provided by Michael Kuska (NEB Organic Synthesis Division). Fast-flow Ni-agarose beads were from Qiagen or NEB. The T7 expression vector pET21b with C-terminal 6× His tag was originally purchased from Novagen (NdeI-XhoI). 5hmdCTP/dGTP/dATP/dTTP mix was purchased from Zymo Research. FPLC DEAE and Heparin columns (5 mL) were purchased from GE HealthCare or Cytiva.

### Endonuclease assays

For restriction of modified DNA, Dam^+^ pBR322 and λ DNA were used. In some cases, we also used M.EcoGII-modified pBR322 (Dam^−^), with all modified adenine bases except in polyA tracks. Restriction buffers used were: NEB buffer 2.1 (medium salt, 50 mM NaCl) or CutSmart buffer (with 50 mM potassium acetate). Endonucleases: we usually perform an enzyme titration in endonuclease activity assays; enzyme concentrations are indicated in each digestion. Buffer compositions in 1× NEB restriction buffers: buffer 1.1 (10 mM Bis-Tris-Propane-HCl, 10 mM MgCl_2_, 100 μg/mL BSA or recombinant albumin, pH 7.0 at 25°C); buffer 2.1 (50 mM NaCl, 10 mM Tris–HCl, 10 mM MgCl_2_, 100 μg/mL BSA or recombinant albumin, pH 7.9 at 25°C); buffer 3.1 (100 mM NaCl, 10 mM Tris–HCl, 10 mM MgCl_2_, 100 μg/mL BSA or recombinant albumin, pH 7.9 at 25°C); CutSmart buffer (50 mM potassium acetate, 20 mM Tris-acetate, 10 mM magnesium acetate, 100 μg/mL BSA or recombinant albumin, pH 7.9 at 25°C). For restriction digestions in different divalent cations, a medium salt buffer (50 mM NaCl, 20 mM Tris–HCl, pH 7.5) was supplemented with divalent cations in 0.1, 1, and 10 mM final concentrations.

### Synthetic oligos with modified and unmodified GATC sites

Single-stranded DNA oligos were synthesized by the NEB organic synthesis division:

Top strand Gm6ATC (top M+).

5′/56FAM/ACTCATGCAGGCATGCAGG/m6A/TCGCAGTCAGATTTATGTGTCATATAGT

ACGTGATTCAAG-3′.

Bottom strand Gm6ATC (bottom M+).

5′-CTTGAATCACGTACTATATGACACATAAATCTGACTGCG/m6A/TC CTGCATGCCTGCATGAGT-3′.

Top strand GATC (unmodified, top M−).

5′/56FAM/ACTCATGCAGGCATGCAGGATCGCAGTCAGATTTATGTGTCATATAGTACGTGATTCAAG-3′.

Bottom strand GATC (unmodified, bottom M−).

5′-CTTGAATCACGTACTATATGACACATAAATCTGACTGCGATCCTGCATGCCTGCATGAGT-3′.

Duplex oligos abbreviation:

Fully modified = M+ top/M+ bottom = M+/M+.

Unmodified = M− top/M− bottom = M−/M−.

Hemi-modified = M+ top/M− bottom = M+/M−.

Hemi-modified = M− top/M+ bottom = M−/M+.

Duplex oligos were digested by DpnI (2 U), MboI (5 U), FcyTI (0.1 μg), Ahi29725I and Apa233I (1 μg) in NEB buffer 2.1 at 37°C for 1 h. For HhiV4I digests, reactions were carried out in NEB buffer 2.1 supplemented with 1 mM MnCl_2_ (this enzyme is a Mn^2+^-dependent REase; see below).

#### Protein expression and purification

C2566 (Dam^+^) competent cells (cloning grade) were provided by NEB. ER2948 (Dam^−^) competent cells were prepared by a modified rubidium chloride method. *E. coli* cells were cultured at 37°C to mid-log phase, and IPTG was added to the culture at 0.5 mM final concentration for protein production (at 18°C overnight). Cells were lysed by sonication in chitin column buffer or Ni-agarose column buffer. Clarified cell lysates with over-expressed proteins (target protein-intein-CBD) or C-terminal 6× His-tagged proteins were loaded onto chitin or Ni-agarose columns, respectively, for affinity purification. The protein purification protocols were used as recommended by the manufacturers. In some cases, the partially purified proteins were further purified by chromatography through DEAE (flow-through to remove nucleic acids at 0.3 M NaCl concentration) and Hi-Trap Heparin (5 mL). BigDye^®^ Terminator v3.1 Cycle Sequencing Kit was purchased from Thermo-Fisher (Applied Biosystems). Restriction gene inserts in plasmids were sequenced to verify the correct sequences. Dam^+^ pBR322 DNA fragments after restriction digestion were sequenced to determine the cut sites. DNA sequence edits were carried out using DNAStar or Geneious software packages. BlastP searches in the GenBank and UniProtKB databases were performed using the respective web servers. NCBI Pfam and conserved domains were used to visualize protein domains of REase homologs.

### Plasmid preparation and transformation

Plasmid mini-prep kits and competent cells were provided by NEB. Plasmid mini-preparation and bacterial transformation were done according to the manufacturer’s recommendation.

#### Clustering locus-specific annotations analysis

For clustering locus-specific annotations (CLANS) analysis, the homologs of the five groups of wH-containing enzymes (sequences of the reference proteins are listed in Supplementary material) were obtained by blast (BlastP, default settings in the UniProtKB website) using the UniProtKB reference proteomes and Swiss-Prot database. A total of 1,258 wH fusion endonuclease homolog proteins were analyzed. The resulting homolog protein fasta files were combined and subjected to CLANS analysis using the MPI Bioinformatics Toolkit. The result of the CLANS analysis is visualized with the CLANS Java application (Frickey and Lupas, 2004). CLANS analyzes all-against-all pairwise sequence similarities to establish relationships within a protein family. In the CLANS network figure, each node (a small colored dot) represents a full-length protein (or wH domain in the lower box), and the line connects two proteins/domains that share sequence similarities. The lengths of the lines represent the degree of sequence similarities, with short lines representing close similarities and vice versa. Each node is the same size, but the size of the color “blob” is related to the number of nodes clustered together.

### Phylogenetic analysis

To perform phylogenetic analysis, we first built a hmm (hidden Markov model) profile with the wH domains of the six representative wH-containing enzymes (note: wH domains only, not full-length proteins). The resulting profile was used to search homologs using hmmsearch (HMMER v3.1b2) on the combined fasta file (see CLANS analysis). We extracted the wH domain in each homolog protein and performed multiple alignments with the five representative wH domains using MAFFT (v7.508). The maximum likelihood tree was constructed using RAxML (v8.2.12) with option -f a to enable rapid bootstrapping for 100 times. Other options used were -p 1237, −x 1,237, −m PROTGAMMAAUTO. The best-scoring maximum likelihood tree with bootstrap values was visualized with iTOL.^1^

### Proteome analysis

The four samples (10 μg each) were digested with trypsin using S-Trap micro-columns as recommended by the manufacturer (PROTIFI). LC–MS/MS of the digests was conducted on the Thermo Orbitrap Exploris, with two injections made per sample. Data were searched using Proteome Discoverer v2.5 against a combined FASTA database containing the Uniprotkb_*E. coli*-B strain proteins and the four winged helix (wH) endonuclease protein sequences (FcyTI, HhiV4I, Ahi29725I, and Apa233I). Results are presented in 8 Excel spreadsheets, one file for each injection (see raw data on the proteome of the purified proteins). The most confident protein ID is determined by Score Sequest HT (the last column in each table—the higher the score, the more confident the identification). Proteins identified are reported with >1 unique peptide per protein and a 1% false discovery rate.

## Results

### Bioinformatic screen of wH domain endonucleases

**Figure 2:**
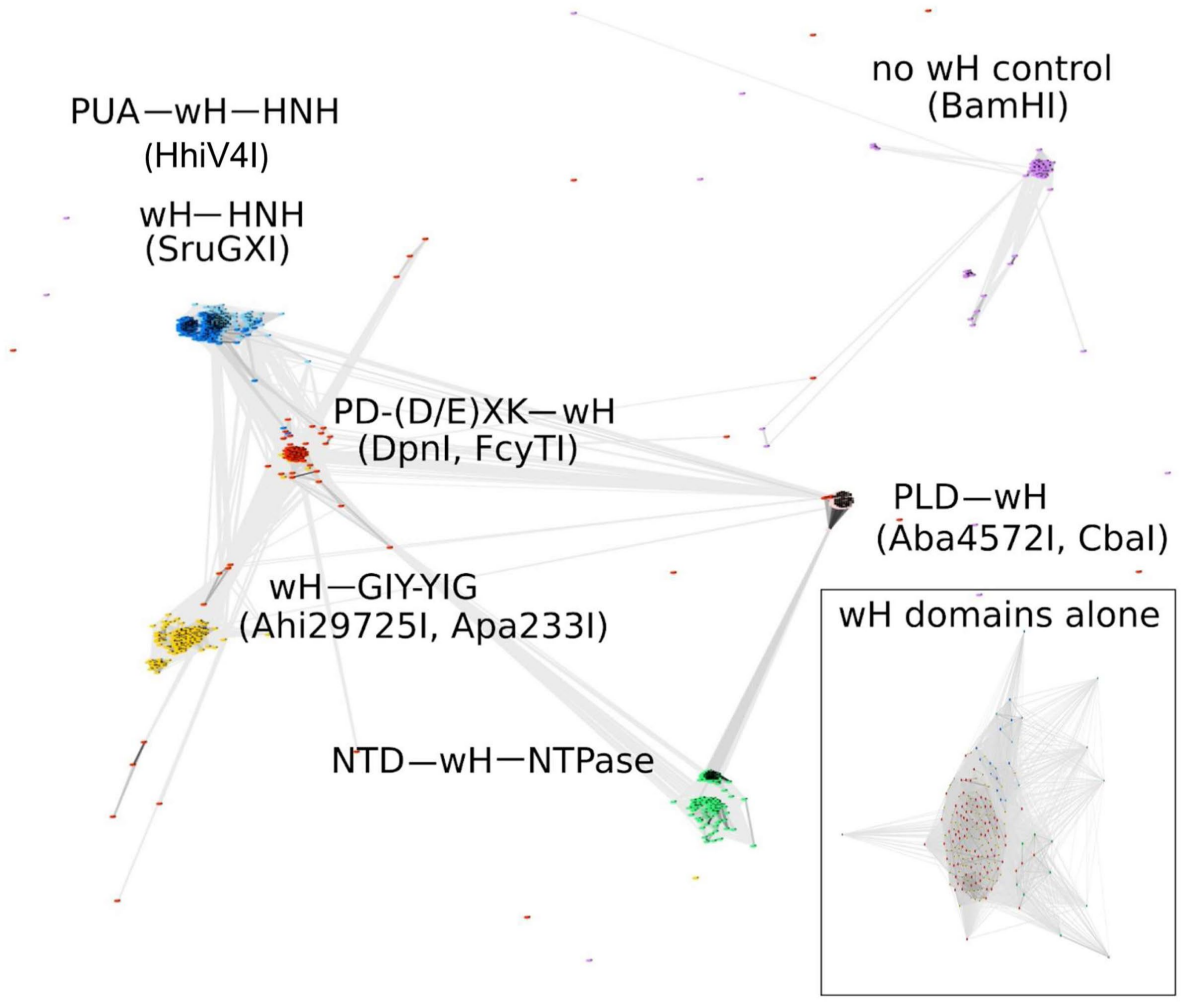
CLANS analysis of the full-length wH fusion proteins with PD-(D/E)XK (marked as red), PUA and HNH (dark blue), HNH (light blue), GIY-YIG endonuclease domains (yellow), and NTD (N-terminal domain)-NTPase (green) found in this study, with BamHI and related isoschizomers as a control group (purple). BOX: CLANS analysis of the wH domains alone, color-coded as in full-length proteins.

Some known wH domains are adenine methylation (6mA)-specific. This notion was originally suggested by the demonstration of 6mA specificity of the DpnI wH domain and further strengthened by the observation of adenine specificity of the wH domain of HHPV4I (Lu et al., 2023), which was reported when this study was being finalized. In the hope of finding new adenine methylation-specific endonucleases, we searched for fusions of wH domains with endonuclease domains known to play roles in R-M (Pingoud and Jeltsch, 2001). Apart from additional PD-(D/E)XK—wH and PUA—wH—HNH endonucleases, we also identified fusion proteins with a wH—HNH, wH—GIY-YIG, and PLD—wH architecture. In addition, we found PD-(D/E)XK—wH—NTPase cases that can be considered as fusions of a DpnI-like protein with an McrB-like NTPase (GTPase/ATPase) domain and NTD—wH—NTPase cases, apparently without a nuclease domain and with an N-terminal domain of unknown function. One additional example is the wH—Mrr catalytic domain (PD-QXK)—NTPase fusion endonuclease. For this study, we concentrated on the NTP-independent enzymes with wH and endonuclease domains. To better understand the sequence relationships between the new wH fusion proteins, we carried out a CLANS (Frickey and Lupas, 2004) analysis. CLANS determines all-against-all pairwise sequence similarities to establish relationships within a protein family. It is not intended to find sequence motifs within protein sequences, which are better detected using other software. In CLANS analysis of the full-length proteins [with BamHI (GGATCC) and related enzymes as a control], the wH domain-containing endonucleases segregated clearly into separate groups, driven by the sequence similarity between endonuclease domains of the same group (Figure 2). However, when we limited the CLANS analysis to the wH domains alone, the segregation into groups according to the endonuclease domain was not so clear. In particular, wH domains from PD-(D/E)XK and GIY-YIG endonucleases were fully intermingled, possibly suggesting multiple separate fusion events (Figure 2, box). The finding suggests that fusions of the same type may have arisen independently several times in evolution. Nevertheless, much of the diversity of the new fusion proteins is clearly due to divergent evolution. This is also supported by the phylogenetic tree of the wH domains (Supplementary Figure S1). Note, however, that bootstrap values for most branches of the tree are very low, making the tree very tentative overall.

### Rare occurrence of methyltransferase genes in the immediate genetic neighborhood of the new fusion proteins

wH proteins frequently bind DNA, but only some of them are methylation-dependent (e.g., DpnI), whereas others are not (e.g., FokI). If the new wH fusion proteins were modification-specific, they should occur as stand-alone enzymes. Otherwise, they should be associated with a host genome protecting DNA methyltransferase of any type (N6mA, N4mC, C5). Typically, such a methyltransferase would be located in the immediate genomic neighborhood of the endonuclease, so that the entire system could work as a defense island (Makarova et al., 2011). To test for possible association with DNA methyltransferases, we inspected the genomic neighborhoods of over 1,000 wH fusion proteins. In 87% of cases, none of the three genes adjacent upstream or downstream to the wH fusion gene encoded a DNA methyltransferase, suggesting that most of the new wH fusion proteins acted as stand-alone endonucleases, possibly as type IV restriction systems.

### Likely specificity of at least some of the wH fusion proteins for 6mA in the GATC context

Adenine methylation in bacteria occurs frequently in the GATC context i.e. the target sequence of the Dam methyltransferase (MTase) (Marinus and Casadesus, 2009), which is widely distributed in bacteria because of its diverse house-keeping roles, including DNA replication (Boye and Lobner-Olesen, 1990) and mismatch repair (Au et al., 1992; Josephs et al., 2015). Hence, it was likely that the putative MDREs with the wH domain might detect adenine methylation in this sequence context. This idea was further supported by the precedent of the wH domain in DpnI, which is known to be specific for adenine methylation in the Dam context (with some leeway for the outer bases S6mATS, where S is G or C). Inspection of the crystal structure of the DpnI domain in complex with target DNA revealed that the same residues contribute to both the methyl binding pocket and the sequence specificity, suggesting that methyl sensing and detection of the G6mATC target sequence are intricately linked (Mierzejewska et al., 2014). Large-scale analysis of the wH domain fusion proteins showed that the motif for 6mA and GATC recognition (see arrows in Figure 1D), or closely related motifs, were present in approximately 10% of the new fusion proteins. With the exception of SruGXI as a representative of the wH-HNH endonucleases, we selected for further characterization the fusion proteins that had the motif for 6mA and GATC specificity. Such fusion proteins are very likely to recognize m6A in the GATC context. The reminder is that stand-alone wH fusion proteins are likely to recognize modified DNA (otherwise they would be toxic to the host). However, it is currently unclear whether the modification is m6A, and, if so, whether the sequence context is GATC.

### Avoidance of genomic conflict

Inspection of a 10 kb interval around genes encoding the new fusion proteins revealed association with methyltransferases in some cases. PD-(D/E)XK—wH domains co-occurred with predicted C5 methyltransferases in 40 cases. In some cases, they also co-occurred with a predicted 4mC (N4mC) or 6mA MTase directly adjacent to it. In these cases (e.g., *Bacteroidota* bacterium isolate CP064983.1, *Moraxella ovis* strain CP011158.1), the putative DNA MTase has been inactivated by a frame shift. The PUA—wH—HNH and wH—HNH co-occurred in 47 cases with Eco57I-like MTases (of type IIG R-M-S fusion enzymes). These MTases are predicted to be 6mA MTases, with CTGAAG (site of methylation underlined) as the target sequence. In the case of the wH—GIY-YIG endonucleases, we found four instances of an EcoRI-like adenine MTase nearby. These MTases are expected to methylate GAATTC. Finally, for the PLD—wH endonucleases, we detected 17 cases of proximity to EcoEI-like (GAGN_7_ATGC) or EcoR124I-like (GAAN_6_RTCG) type I methyltransferases, also causing no conflict. Genetic conflict would not be expected in any of these cases if the new wH fusion proteins had specificity for 6mA in the GATC context.

Next, we looked for possible genetic conflicts on a genome-wide scale, assuming that the new wH fusion proteins were specific for m6A in a GATC context. Three types of such conflict are conceivable. First, a frequent adenine methyltransferase, such as M.EcoGII, may methylate adenine to m6A in GATC, among many other contexts. Second, a Dam-like methyltransferase may specifically modify GATC sequences. Finally, there are also methyltransferases that methylate target sequences that are longer than GATC but include the GATC site in the recognition sequence. We searched for cases of such potential conflict, scanning the entire genome, not just the genomic neighborhoods. Overall, less than 100 cases of potential conflict were identified (Supplementary Tables S1–S3) for over 1,000 wH fusion proteins. Most of the wH fusion proteins in potential conflict are stand-alone enzymes without an associated methyltransferase. Genomic conflict could be avoided if these proteins recognized modified DNA containing a mark other than m6A in the GATC context (i.e., either another methylation type or m6A in a different sequence context). Alternatively, conflict may be avoided or mitigated by tight expression control of the endonuclease or the methyltransferase.

### Selection of wH fusion proteins for experimental characterization

Four types of endonuclease domains are noted in wH domain fusions: (1) DpnI-like PD-(E/D)XK endonuclease; (2) HNH endonuclease domain; (3) GIY-YIG endonuclease domain; and (4) PLD family endonuclease domain. An NTD-wH-NTPase fusion is usually paired with another endonuclease subunit, such as McrC-like catalytic subunit, which is not discussed in detail here. We have not studied evolutionary relationships within each endonuclease family since the endonuclease families have been the subject of numerous review articles and research papers (Mehta et al., 2004; Grazulis et al., 2005; Pingoud et al., 2005; Dunin-Horkawicz et al., 2006). For experimental characterization, we chose representatives of the PD-(D/E)XK—wH (FcyTI), PUA—wH—HNH (HhiV4I), wH—HNH (SruGXI), wH—GIY-YIG (Ahi29725I, Apa233), and PLD—wH (Aba4572I, CbaI) fusions for further experimental characterization. In the case of the PUA—wH—HNH architecture, many additional candidate MDREs were tested in *E. coli* cells only. An attempt to purify an NTD—wH—NTPase/McrC-like subunit fusion protein was unsuccessful. Therefore, we focused this study exclusively on single-chain proteins.

### The wH fusion endonucleases exhibit Dam^+^-dependent toxicity in *E. coli* cells

**Figure 3:**
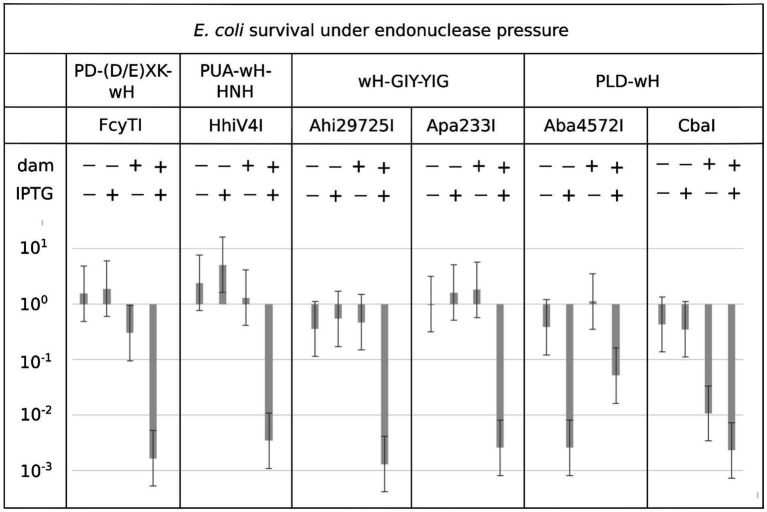
Toxicity of selected wH domain fusion endonucleases to a Dam^+^ but not a Dam^−^ host. Expression vectors containing open reading frames for putative MDREs or empty plasmid (50 ng) were transformed into Dam-positive C2566 (+) or Dam-negative ER2948 (−) *E. coli* cells, with (+) or without (−) IPTG induction. The reduction in colony counts for expression plasmid compared to the empty vector, plotted on the ordinate, is a measure of toxicity.

Endonuclease toxicity is a good proxy for restriction in bacterial cells (Heitman and Model, 1990; Fomenkov et al., 1994). If the putative MDREs were specific for Dam-methylated DNA, they should be more toxic to Dam^+^ (C2566) than to Dam^−^ (ER2948) *E. coli* cells. We tested this prediction with our IPTG-inducible expression constructs, both under basal (no IPTG) conditions and under induction conditions (0.5 mM IPTG). Since Dam^−^ competent cells were roughly an order of magnitude less competent than Dam^+^ cells, we avoided direct comparisons of transformation efficiency between Dam^+^ and Dam^−^ cells. Instead, we quantified the reduction in colony counts for transformations with expression plasmids compared to colony counts with an empty vector. With the exception of the Aba4572I expression construct, the plasmids for all other endonuclease-containing clones caused a reduction in colony count by two to three orders of magnitude compared to the empty vector control in Dam^+^ cells under induced conditions (Figure 3). Aba4572I endonuclease may have strong non-specific endonuclease activity since it is toxic in a Dam-deficient strain under IPTG induction. The toxicity appeared to be less severe in Dam^+^ cells. This result is not well understood. If the Aba4572I outlier is disregarded, the experimental results indicate that the wH fusion endonucleases display typical “restriction” on Dam^+^ host but not Dam^−^ cells. Whether this *in vivo* “restriction” was caused by tight binding to modified sites to inhibit replication or endonuclease cleavage of modified DNA remains to be investigated.

### PD-(D/E)XK—wH endonucleases

**Figure 4:**
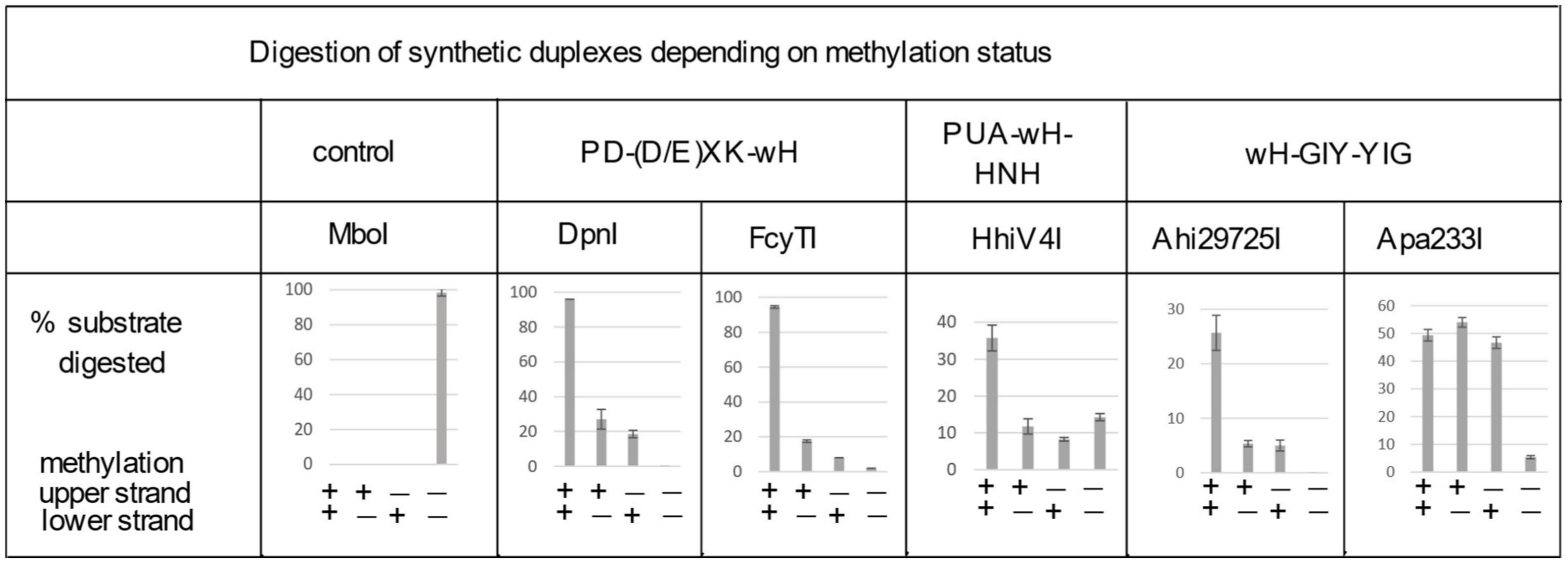
Digestion of synthetic DNA oligoduplexes depending on top and bottom strand methylation status in the Gm6ATC sequence context. 5 U of MboI (GATC) and 2 U of DpnI (Gm6ATC) were used as controls. FcyTI input was 0.1 μg protein (~3 U) (higher concentrations could obscure the digestion of hemi-modified duplex oligos). Duplex oligos and protein concentration in restriction digests: The duplex oligos concentration is approximately 18 nM (60mer, 21 ng in 30 μL reaction volume). The protein concentration is calculated below in the 30 μL reaction volume: (1) Ahi29725I protein (dimer) MW = 24.62 × 2 = 49.24 kDa, Ahi29725I, 1 μg = ~677 nM. (2) Apa233I protein (dimer) MW = 24.17 × 2 = 48.34 kDa, Apa233I, 1 μg = ~690 nM. (3) HhiV4I protein (dimer) MW = 44.67 × 2 = 89.34 kDa, HhiV4I, 1 μg = ~373 nM. (4) FcyTI protein (dimer) MW = 30.76 kDa × 2 = 61.52 kDa, FcyTI 0.1 μg = 54 nM.

As representatives of the PD-(D/E)XK—wH family, we selected Psp4BI and FcyTI (GenBank accession numbers WP_102090895 and WP_094411979). The two enzymes have 58.4 and 58.7% amino acid (aa) sequence identity to DpnI, respectively. Psp4BI was chosen because the source organism is psychrophilic, suggesting that the enzyme might be susceptible to heat inactivation, which would be desirable for biotechnological applications. The synthetic genes with *E. coli* optimized codons were cloned into pTXB1 in fusion with intein and CBD (chitin-binding domain) and expressed in the Dam-deficient T7 expression strain. The two enzymes were affinity purified on a chitin column and released from the column by DTT-triggered cleavage. The yield of Psp4BI was low due to poor expression of the fusion protein (Psp4BI-intein-CBD) (not shown); partially purified Psp4BI gave rise to a partial digestion pattern that was retained after 4 h at 25–37°C. The low activity could be caused by a low enzyme concentration or inhibition by some impurities in the preparation. By contrast, purified FcyTI was active on Dam^+^ pBR322, pUC19 (HindIII-linearized), and phage λ DNA (Supplementary Figures S2A,B). FcyTI-specific activity was determined as approximately 32,000 U/mg protein in buffer 2.1. FcyTI could be inactivated by heating at 65°C for 30 min, which is a useful enzyme property (Supplementary Figure S3). FcyTI endonuclease was originally found in the genome of *Flavobacterium cyanobacteriorum*, which grows at 20–30°C. Due to its better biochemical properties, FcyTI was used for the *in vivo* toxicity study (Figure 3) and for the digestion of modified oligos (see Figure 4). The FcyTI expression plasmid showed over a 1,000-fold reduction in transformation efficiency in the Dam^+^ T7 strain compared to a Dam^−^ host (Figure 3). FcyTI could be over-expressed only in the Dam^−^ T7 expression strain. Run-off sequencing demonstrated that the enzyme was able to cleave within the Gm6A↓TC recognition sequence (Supplementary Figure S4). The purified 6× His-tagged FcyTI shown in Supplementary Figure S5 was used for the digestion of modified or hemi-modified oligoduplexes.

To compare the activity of the enzyme toward fully methylated, hemi-methylated, and non-methylated DNA, we digested synthetic DNA oligoduplexes and quantified substrate and product amounts after restriction digestion by capillary electrophoresis (CE) (Figure 4). The results showed that FcyTI was most active on fully methylated DNA but also had partial activity on hemi-methylated DNA, similar to DpnI. No digestion product was detected for non-methylated DNA. MboI was used as a control and digested only unmodified GATC oligos. Fully and hemi-modified substrates were resistant to MboI restriction (Figure 4). The original CE digestion results are shown in raw data (PeakScan analysis of CE peaks). Consistent with the duplex oligos digestion, unmodified pUC19 (HindIII-linearized) or phage DNA were poorly cleaved by FcyTI (Supplementary Figure S2), although weak activity was observed on Dam^−^ pUC19, probably due to the high enzyme concentration.

### PUA-wH-HNH fusion endonuclease HhiV4I

6× His-tagged HhiV4I (see Supplementary Figure S5) was subjected to three-step chromatography (Ni-agarose column, DEAE column, and Heparin agarose column). Compared to the recently published paper on the same enzyme (Lu et al., 2023), two additional chromatography steps were used (DEAE and Heparin columns). Unfortunately, the Heparin agarose chromatography step was less efficient for purification than is typical for other DNA-binding proteins because HhiV4I was in the flow-through and did not bind to the Heparin column, as would be expected for a typical nucleic acid-binding protein. As a result, HhiV4I was not purified to homogeneity (Supplementary Figure S5). Mass spectrometry analysis of the contaminations identified, among other proteins, *E. coli* exonuclease (Exonuclease VII, Exo VII) as a minor contaminant (see raw data for the HhiV4I mass spectrometry study). Exo VII cleaves single-stranded DNA (ssDNA) from both the 5′ → 3′ and 3′ → 5′ directions. This enzyme is not active on linear or circular dsDNA. The contaminating exonuclease would not likely interfere with major cut site determination, but it may interfere with minor cut site(s) by removing a few nucleotides for the cleaved ends by HhiV4I if the overhang is single-stranded.

**Figure 5:**
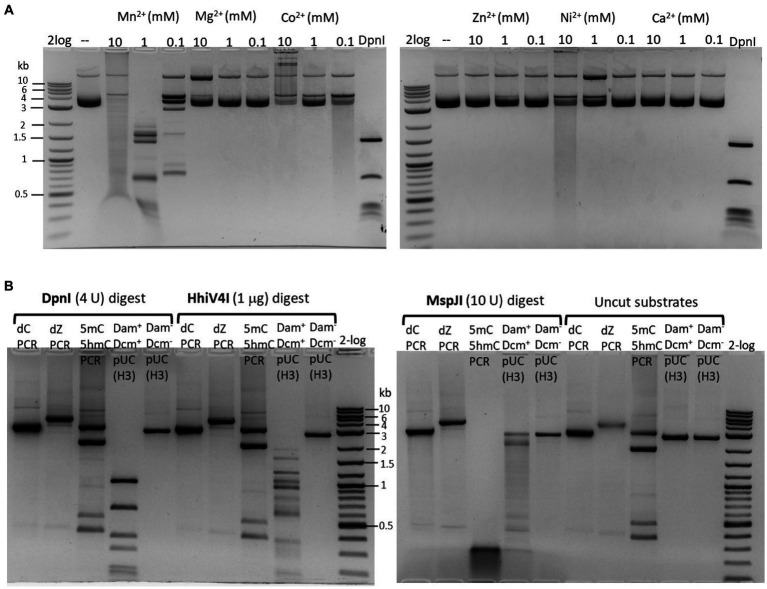
**(A)** Divalent cation requirement for HhiV4I restriction activity. Divalent cations were supplemented in a medium salt buffer (50 mM NaCl, 10 mM Tris–HCl, pH 7.5) at 10, 1, and 0.1 mM final concentration, respectively. HhiV4I (1 μg) was used in the digestion of pBR322 (Dam^+^, 1 μg) at 37°C for 1 h. Mn^2+^ supports HhiV4I activity as the preferred cofactor. In 10 mM Mg^2+^ buffer, the enzyme showed partial nicking activity. In Co^2+^ and Ni^2+^ buffers, the enzyme showed weak but detectable activity. **(B)** HhiV4I incubation with 5mC−, 5hmC−, or dZ− (2-aminoadenine, 2,6-aminopurine) modified PCR DNAs. DpnI, MspJI, and HhiV4I digests were carried out in 1× NEB buffer 2.1, CutSmart buffer, and B2.1 plus 1 mM Mn^2+^, respectively. Modified and unmodified DNA substrates: (1) dC regular PCR-unmodified (2.9 kb), (2) dZ PCR (2-aminoadenine modified, 4 kb), (3) a mixture of 5mC (2 kb) and 5hmC (2.9 kb) PCR products, and two minor PCR products (0.4–0.5 kb), (4) HindIII (H3)-prelinearized pUC19 (Dam^+^ Dcm^+^, 2.7 kb), (5) HindIII (H3)-prelinearized pUC19 (Dam^−^ Dcm^−^, 2.7 kb). DpnI and HhiV4I digested linear Dam^+^ pUC19 DNA. MspJI digested 5mC/5hmC modified PCR DNA and Dcm^+^ linear pUC19 (MspJI site 5(h)mCNNR, Dcm-methylated sites C5mCWGG).

The partially purified 6× His-tagged HhiV4I was used for HhiV4I characterization. Consistent with the findings of Lu et al. (2023), we observed that HhiV4I was much more active in the presence of Mn^2+^ ions than in the presence of other divalent metal cations (Figure 5A). HhiV4I showed weak DNA-nicking activity in Mg^2+^ buffer.

**Figure 6:**
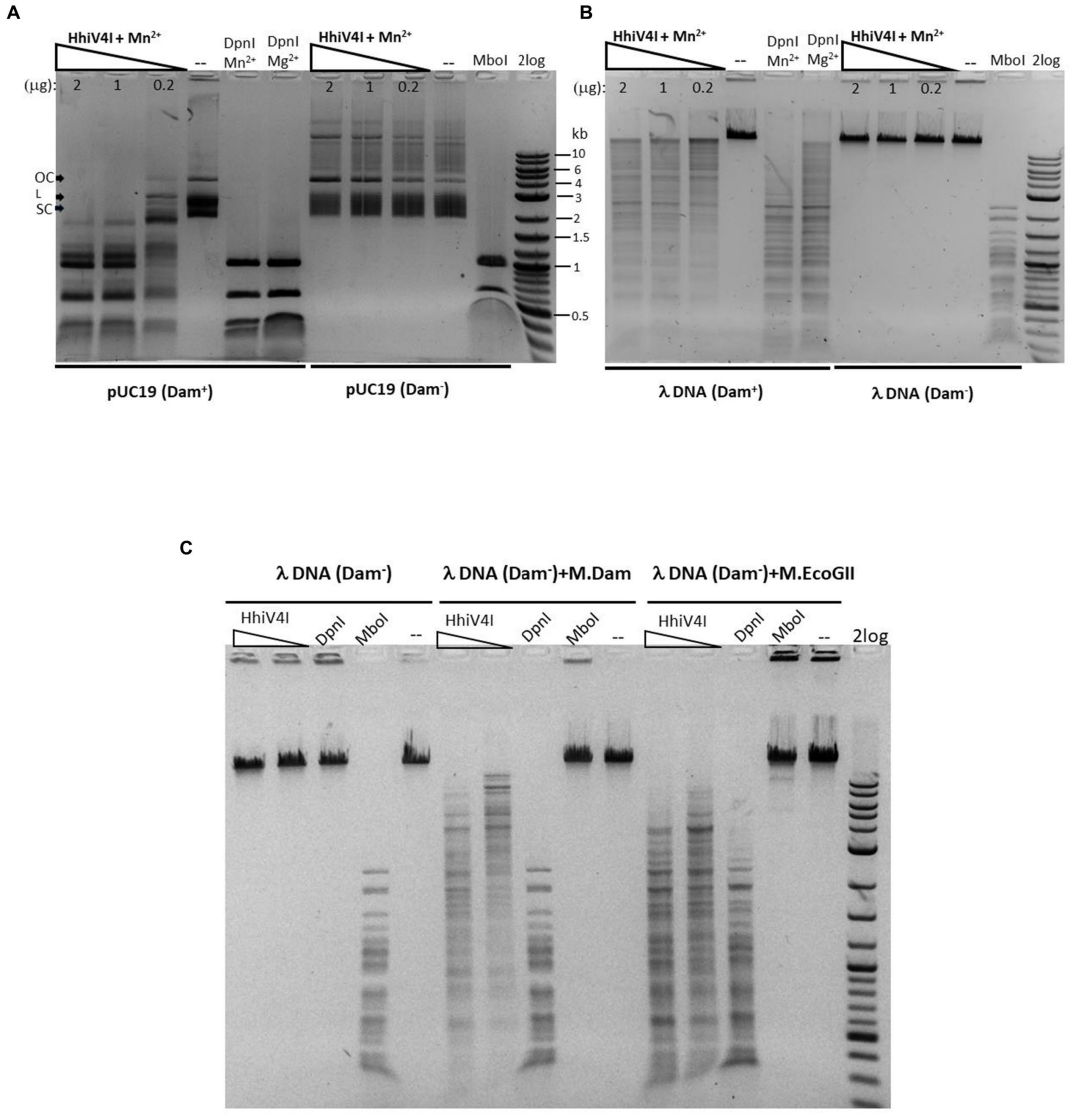
Methylation dependence of HhiV4I endonuclease. **(A)** Restriction digests of 1 μg of Dam^+^ or Dam^−^ pUC19 in Mn^2+^ buffer by HhiV4I (447.7, 223.9, and 44.8 nM, respectively, in 50 μL reaction volume). **(B)** Restriction digests of 1 μg of Dam^+^ or Dam^−^ phage λ DNA in Mn^2+^ buffer by HhiV4I (447.7, 223.9, and 44.8 nM, respectively, in 50 μL reaction volume). DpnI (2 U) cleaves Dam^+^ DNA only. MboI (5 U) cleaves unmodified DNA only. **(C)** HhiV4I digestion (447.7 and 44.8 nM) of 1 μg of *in vitro* modified λ DNA (Dam^−^) by Dam methylase or frequent adenine methylase M.EcoGII in Mn^2+^ buffer in 50 μL reaction volume. Following methylation reactions, the methylases were inactivated by heating at 65°C for 20 min. The methylated DNA was then diluted for restriction digestion in Mn^2+^ buffer.

In agreement with the toxicity experiments (Figure 3) and the results of Lu et al. (2023), we found that the enzyme had higher activity against Dam^+^ than Dam^−^ pBR322, pUC19, λ DNA, and synthetic duplex oligos. If HhiV4I cleaved at or near Dam^+^ sites, its cleavage products should be of similar size as those of DpnI digestion, and discrete bands (as opposed to a smear on the gel) should be observed. In our experiments, we saw only a partial match of fragment sizes, likely due to incomplete digestion (Figure 6A) (see below for the two-site requirement for efficient cleavage). Dam^+^ phage λ DNA was also only partially digested while Dam^−^ λ DNA was not cut at all (λ DNA was partially methylated by the host Dam methylase during rapid phage replication in *E. coli*, unpublished observation) (Figure 6B). When Dam^−^ λ DNA was methylated *in vitro* by Dam methylase or M.EcoGII, the DNA substrates now became cleavable by HhiV4I (Figure 6C), further demonstrating that GATC methylation is required for restriction. M.EcoGII-modified λ DNA appeared to be a slightly better substrate for HhiV4I restriction than Dam-modified λ DNA, indicating that the wH might not be strictly limited to the detection of 6mA in the GATC context.

We could digest non-methylated DNA with excess HhiV4I, suggesting that the dependence of the enzyme on adenine methylation was not absolute. Most restriction enzymes display star activity at high enzyme, high glycerol concentration, or low salt. This conclusion was confirmed with the digestion of synthetic DNA with a defined adenine methylation status. As expected, HhiV4I was most active on fully methylated DNA but had some activity on hemi- and non-methylated DNA (Figure 4). In agreement with the findings by Lu et al. (2023) we did not detect activity of HhiV4I toward PCR products, which contained 5mC or 5hmC instead of C, in conditions conducive to digestion of m6A containing DNA (Figure 5B). Since the PCR products contain 5mC and 5hmC in many different contexts, this result suggests that the enzyme has no activity against methylated or hydroxymethylated DNA, despite the presence of the PUA (SRA-like) domain. This was surprising because it had been shown previously that PUA superfamily REases VcaM4I, SRA-like domain-containing endonuclease TagI, and PvuRts1I restricted DNA containing modified cytosines (Janosi et al., 1994; Pastor et al., 2021). Possible activity against WT T4 [glucosylated(g)-5hmC] modified DNAs remains to be tested. HhiV4I shows no activity on dZ (modified adenine, 2-aminoadenine, or 2,6-diaminopurine)-modified PCR DNA (Figure 5B).

HhiV4I prefers to cut between two G6mATC sites with optimal spacers of 13–27 bp in Dam^+^ pBR322. Shorter spacers of 8–11 bp or longer spacers >42 bp were cleaved more slowly. Run-off sequencing of Dam^+^ HhiV4I DNA confirmed that the enzyme cleaved in the vicinity of but not within the G6mATC sequence, as previously reported (Supplementary Figure S6).

### PUA—wH—HNH and wH—HNH endonucleases

**Figure 7:**
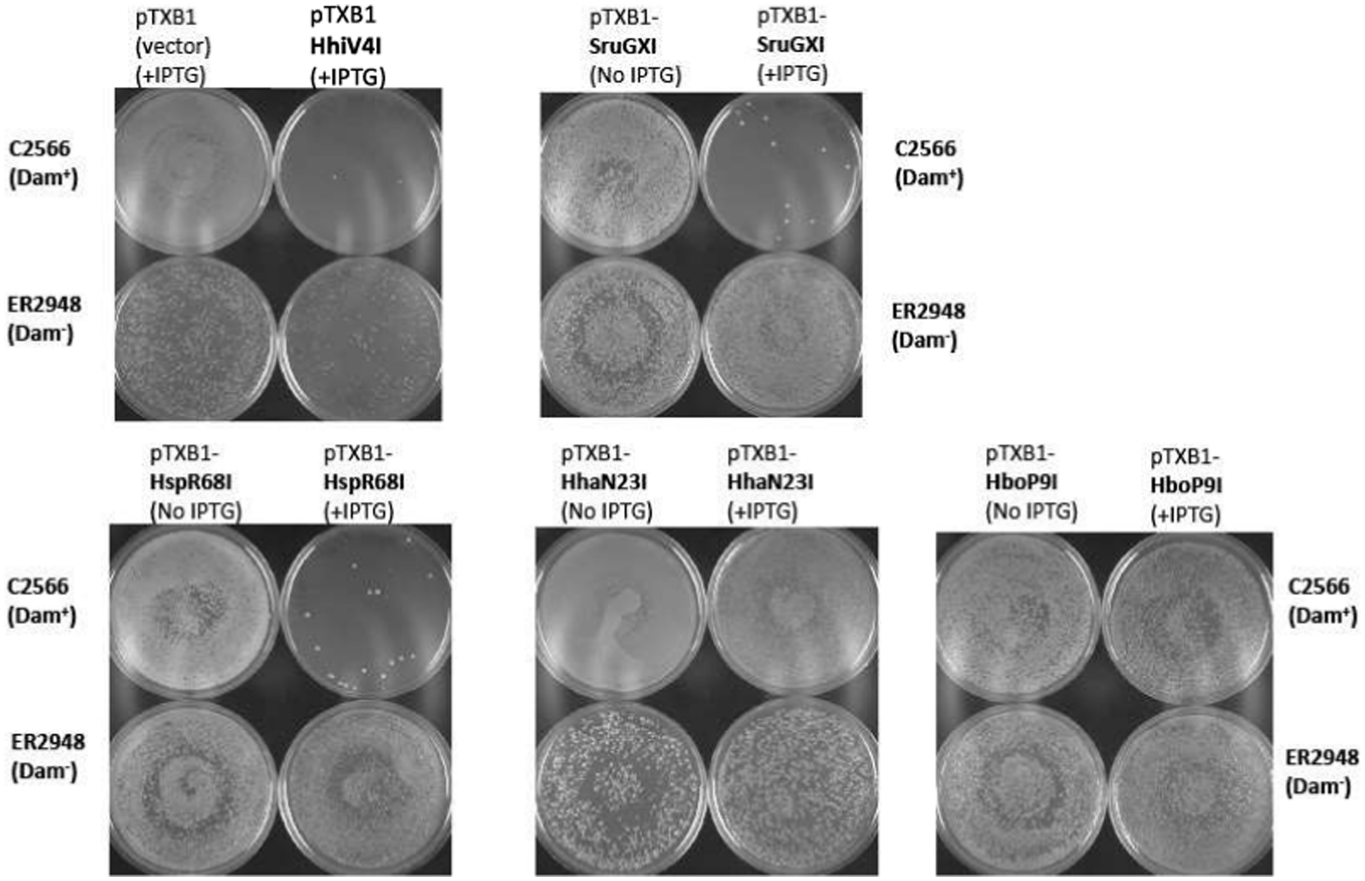
*In vivo* toxicity study: plasmid transfer into C2566 (Dam^+^) and ER2948 (Dam^−^) competent cells by transformation (~50 ng plasmid DNA). Three types of restriction phenotypes were observed: a strong reduction in transformation efficiency due to gene conflict (e.g., HhiV4I, SruGXI, and HspR68I); small colony formation in Dam^+^ hosts presumably due to mild toxicity of the restriction gene (e.g., HhaN23I); and no noticeable change in transformation efficiency (e.g., HboP9I) compared to the empty vector control. The assay was done semi-qualitatively based on visualization of the transformation plates and not quantitatively since the number of colonies was not counted. Toxicity was more apparent with IPTG induction (0.5 mM IPTG in Amp plates). The overall transformation efficiency is lower in the Dam-deficient host.

In contrast to the prophage-encoded HhiV4I, most PUA—wH—HNH enzymes (375–496 aa long) and wH—HNH endonucleases (224–283 aa long) are bacterial/archaeal enzymes. For 15 of these enzymes and HhiV4I as a positive control, we attempted expression in the Dam^−^ *E. coli* cells. Moreover, we analyzed the transformation efficiency into Dam^+^ (C2566) and Dam^−^ (ER2948) cells compared to the empty vector. Restriction activity was examined in the presence of IPTG induction to elevate the genome conflict (Table 1). Some restriction genes, such as HhiV4I and SruGXI, had a strong toxic effect, as detected by a 100–1,000-fold reduction in transformation efficiency in the Dam^+^ host. Other ORFs caused an approximately 10-fold reduction in transformation efficiency, consistent with partial restriction (+/−). The transformation of the HhaN23I gene caused the formation of very small colonies in the presence or absence of IPTG, indicating partial restriction. A few ORF constructs showed no difference in transformation efficiency in the Dam^+^ host, presumably as a result of poor expression or lack of activity (e.g., HboP9I). As a control, the pTXB1 empty vector could be readily transferred into C2566 (Dam^+^) or ER2948 (Dam^−^) cells in the presence of IPTG (Table 1; Figure 7).

**Table 1:** *In vivo* toxicity of PUA-wH-HNH and wH-HNH endonucleases.

GenBank accession number (protein)	*Halobacteria* (archaea) or bacterial strain	Enzyme name	Protein expression level	Toxicity *in vivo*	Activity *in vitro*
1. ARM71120.1	*Haloarcula hispanica* pleomorphic virus 4	HhiV4I (381 aa)	+++	+	+
2. WP_007980235.1	*Haladaptatus paucihalophilus* DX253	HpaD253I (234 aa)	+++	+	−
3. WP_009365977.1	*Halogranum salarium* B-1	Hsa1 (375 aa)	+++	+	−
4. WP_098725488.1	*Natrinema* sp. CBA1119	NspC1119I (480 aa)	+++	+	−
5. WP_049951637.1	*Halostagnicola larsenii* XH-48	HlaX48I (378 aa)	+++	+	−
6. WP_135828390.1	*Halorussus halobios* HD8-83	HhaH883I (224 aa)	+++	+	−
7. WP_103425724.1	*Salinigranum rubrum* GX10	SruGXI (232 aa)	+++	+	ND
8. WP_128477186.1	*Halorussus* sp. RC-68	HspR68I (283 aa)	++	+	ND
9. WP_012944509.1	*Haloterrigena turkmenica* DSM5511	HtuIII (496 aa)	++	+/−	+/−
10. WP_008893710.1	*Haloterrigena salina* JCM13891	Hsa13891I (496 aa)	+++	+/−	+/−
11. WP_126662294.1	*Haloterrigena salifodinae* ZY19	HsaZ19I (496 aa)	+++	+/−	−
12. WP_126597564.1	*Dictyobacter aurantiacus* S27 (G^+^ bacterium)	DauS27I (372 aa)	−	+/−	−
13. WP_009486715.1	*Halobacterium* sp. DL1	HspD1I (230 aa)	−	+/−	−
14. WP_117591244.1	*Haloprofundus halophilus* NK23	HhaN23I (235 aa)	+++	+/− (small colonies)	ND
15. WP_006089674.1	*Natronorubrum tibetense* GA33	NtiG33I (492 aa)	+++	−	−
16. WP_159527272.1	*Halobacterrium bonnevillei* PCN9	HboP9I (230 aa)	+++	−	ND

Protein expression, *in vivo* toxicity, and *in vitro* activity of PUA—wH—HNH and wH—HNH endonucleases. The *in vivo* toxic effect of the restriction gene was measured by transformation into Dam^+^ and Dam^−^ *E. coli* competent cells under IPTG induction. +, strong restriction (100–1,000-fold reduction in Dam^+^ cells), +/−, mild restriction (~10-fold reduction in Dam^+^ cells or formation of sick small colonies), −, no restriction. Protein expression level: +++, 2–10 mg protein per liter of IPTG-induced cells; ++, 1–1.5 mg/L; −, target protein not detected after chitin column purification or in IPTG-induced cell extract. Proteins with 375–496 aa residues are PUA—wH—HNH fusions; proteins below 300 aa residues are wH—HNH fusions. DauS27I is found in the G^+^ bacterium *Dictyobacter aurantiacus* S27. The other enzymes are found in Archaea (*Halobacteria*). ND, not determined. Dam^+^ pBR322 was used for *in vitro* cleavage assays.

Selected enzymes that appeared to be promising as Dam^+^-dependent MDREs were partially purified, and their activity was tested on Dam^+^ pBR322 or λ DNA. The partially purified HtuIII enzyme (GenBank accession number NC_013743, PUA—wH—HNH fusion) shows a low nicking activity in Mn^2+^ or Co^2+^ buffer (Supplementary Figure S7). DNA run-off sequencing of the partially nicked pBR322 indicated that the nick occurred upstream of the Gm6ATC site (top strand nicking only; ↓NGm6ATC-N14-Gm6ATC). The results of wH-HNH and PUA-wH-HNH endonuclease activities and *in vivo* toxicity are summarized in Table 1.

Analogous to HhiV4I, HtuIII also preferred Mn^2+^ or Co^2+^ for catalytic activity, suggesting that both enzymes have a unique metal ion binding site that is different from the typical HNH ββα-metal catalytic domain found in type II REases, homing endonucleases, Cas9, and non-specific endonucleases utilizing Mg^2+^ as a cofactor. The cofactor preferences are similar to the preferences of *E. coli* EcoKMcrA endonuclease and ScoMcrA (Liu et al., 2010).

### wH—GIY-YIG endonucleases

Two wH—GIY-YIG fusion proteins, Ahi29725I (WP_035368356) and Apa233I (WP_026653965), were selected for characterization. The proteins occur naturally in *Acholeplasma hippikon* (ATCC29725 strain) and *Acholeplasma palma* (J233 strain), respectively. *Acholeplasma* are bacteria without cell walls in the Mollicutes class with small genomes (1.5–1.65 mbp). *Acholeplasma* species are found in animals, insects, and some plants in the environment. Some *Acholeplasma* species are pathogenic and can contaminate mammalian cell cultures. We expressed both proteins in Dam^−^ *E. coli* and purified the proteins by chromatography through chitin, DEAE, and Heparin columns. The analysis of the purified proteins on SDS-PAGE is shown in Supplementary Figure S5. Protein mass spectrometry analysis of the purified enzymes showed minimal exonuclease contamination (see raw data for protein composition analysis).

The divalent cation requirement for the Ahi29725I GIY-YIG endonuclease activity was assessed in a medium salt (50 mM NaCl) buffer (Supplementary Figure S8). Ahi29725I is active in Mg^2+^ (1–10 mM) and Mn^2+^ (0.1–10 mM) buffers and partially active in Co^2+^ (1–10 mM) and Ni^2+^ (1–10 mM) buffers in digestion of pBR322 (Dam^+^). It has a nicking activity in Ca^2+^ (10 mM) buffer. To assess the 6mA-dependent restriction activity, Ahi29725I was also assayed on Dam^−^ pBR322 in the presence of Mg^2+^, Mn^2+^, or Co^2+^ in restriction digests. The results showed that the enzyme digested Dam^−^ DNA into a smearing pattern in Mn^2+^ buffer without discrete bands, probably as a result of loss of specificity (data not shown). It is known that type II REases and homing endonucleases (HEases) with the GIY-YIG endonuclease domain preferentially use Mg^2+^ divalent cation as a cofactor. The purified Apa233I showed a similar divalent cation preference as Ahi29725I since it is active in restriction buffers with Mg^2+^ or Mn^2+^ (data not shown).

**Figure 8:**
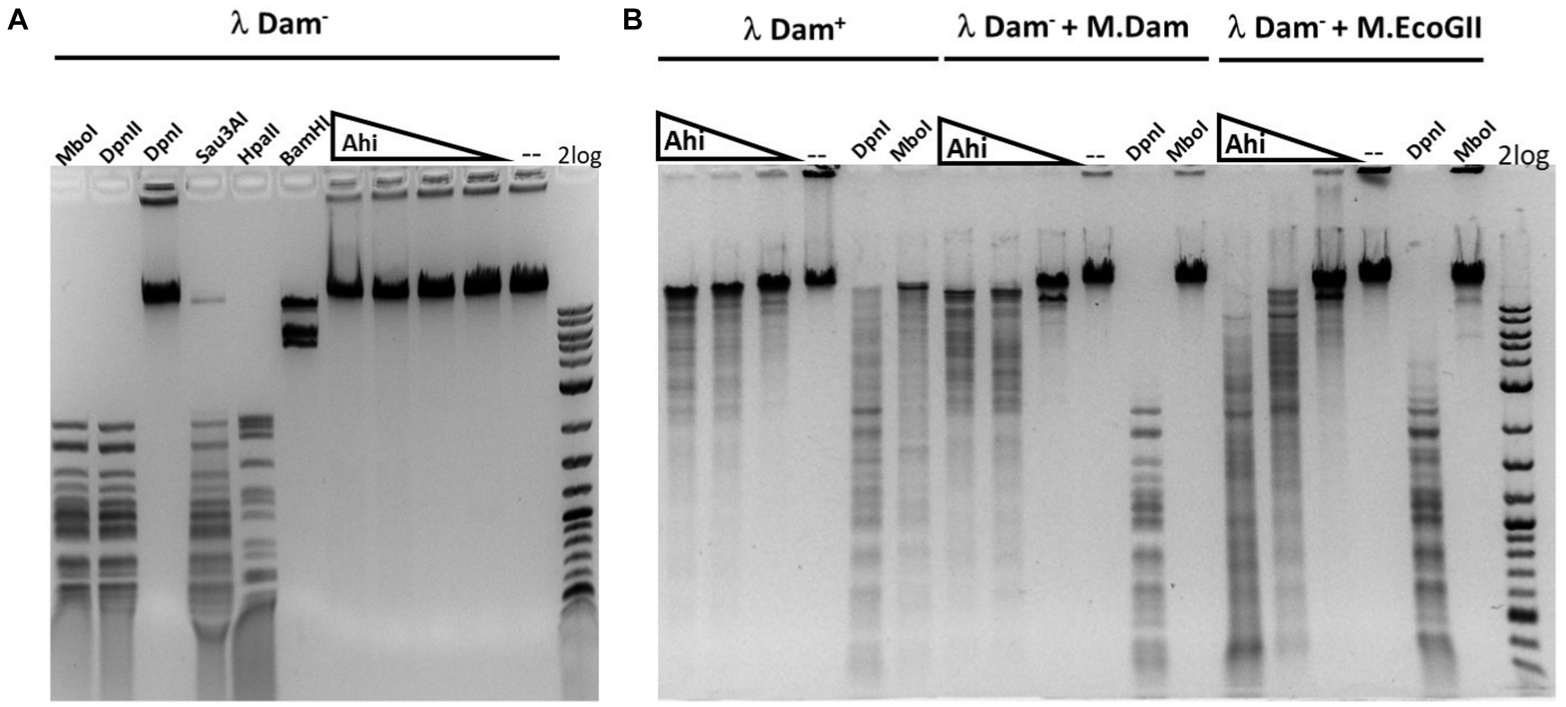
Restriction activity assay on Dam^+^, Dam^−^ λ DNA, Dam^−^ λ DNA further modified by Dam methylase (M.Dam) or M.EcoGII. **(A)** Restriction digestion of Dam^−^ λ DNA with Ahi29725I (Ahi) 10-fold serial dilution: 812.4, 81.2, and 8.1 nM (2, 0.2, and 0.02 μg) of protein incubated with 0.5 μg λ DNA in NEB B2.1 in 50 μL reaction volume at 37°C for 1 h. **(B)** 10-fold serial dilution of Ahi29725I (Ahi) in digestion of Dam^+^ λ DNA, Dam^−^ λ DNA methylated by Dam methylase or by M.EcoGII. λ DNA was only partially modified by *E. coli* host Dam methylase during rapid phage DNA replication. In control digests, MboI and DpnII cleave unmodified GATC sites only; DpnI cleaves Gm6ATC sites only; Sau3AI cleaves GATC sites regardless of m6A methylation. HpaII (CCGG) and BamHI-HF (GGATCC, not affected by Dam methylation) are additional controls.

**Figure 9:**
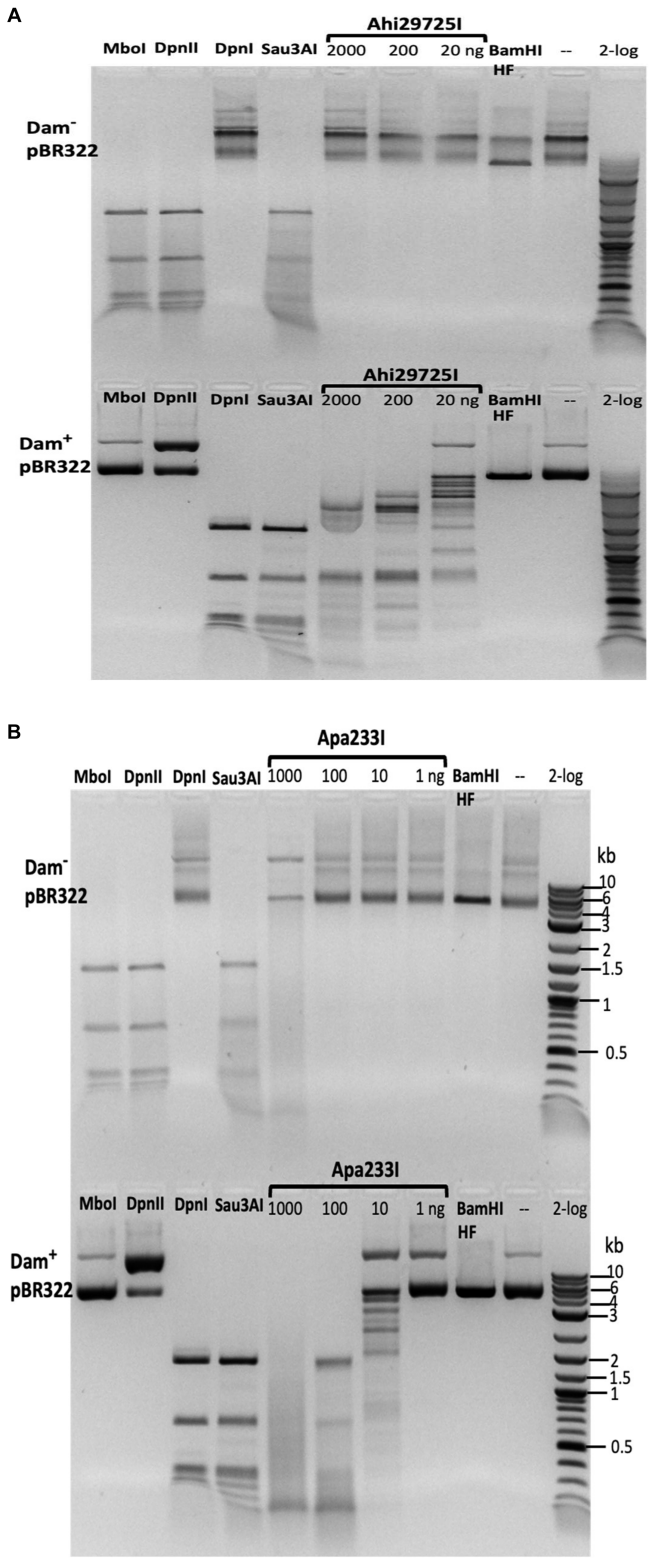
*In vitro* activity of Ahi29725I **(A)** and Apa233I **(B)** on Dam^−^ (top) and Dam^+^ (bottom) DNA. Ahi29725I (~812.4, 81.2, and 8.1 nM, respectively) or Apa233I (~413.7, 41.4, 4.1, and 0.4 nM, respectively) digestion of plasmid DNA (0.5 μg) was done in NEB B2.1 at 37°C for 1 h in a 50 μL reaction volume. In control digests, MboI and DpnII cleave unmodified GATC sites only; DpnI is unable to cut Dam^−^ DNA. Sau3AI cleaves GATC sites regardless of 6mA methylation. BamHI-HF (GGATCC) was used as an additional control. Apa233I showed non-specific endonuclease activity at high enzyme concentrations (~1 μg/0.4 μM). A smeared pattern was detected in both Dam^+^ and Dam^−^ pBR322.

The purified Ahi29725I enzyme was assayed on Dam^+^ and Dam^−^ λ DNA to test modification dependence (Figure 8). Dam^−^ λ DNA was also methylated by Dam methylase (M.Dam) or EcoGII frequent adenine methylase (M.EcoGII) in the test tube and used for Ahi29725I digestions. The Ahi29725I endonuclease generated a partial digestion pattern on Dam^+^ λ DNA. It showed no cleavage activity on Dam^−^ λ DNA, indicating restriction activity dependent on Dam modification. When Dam^−^ λ DNA was methylated *in vitro* by Dam methylase or M.EcoGII, the modified substrates now became cleavable by Ahi29725I (Figure 8). In control digestion, MboI, DpnII, and Sau3AI are able to cleave Dam^−^ λ DNA, but DpnI cannot. Similarly, Ahi29725I and Apa233I endonucleases are also active on Dam^+^ pBR322 and inactive on Dam^−^ pBR322 (Figure 9). However, high enzyme concentrations of Apa233I resulted in non-specific digestion (smearing) of Dam^−^ DNA. The finding was attributed to the non-specific activity on unmodified DNA, since most restriction enzymes display star activity at high enzyme, high glycerol concentration, or low salt.

The Ahi29725I and Apa233I digested pBR322 (Dam^+^) DNA was subjected to run-off sequencing with primers annealing near the Gm6ATC sites. Cleavages occurred outside the recognition sequence, at a variable distance from the site of methylation (i.e., Ahi29725I cleaves G6mATC at N_1–23_) (Supplementary Figures S9, S10). Ahi29725I and Apa233I have limited, if any, preference for cleavage at NN/RN and NN/GN sites, respectively (Supplementary Figure S11), which would have to be attributed to endonuclease sequence preferences.

To test whether Ahi29725I catalyzed DNA cleavage could be directed by adenine methylation in addition to the G6mATC sequence context, we digested M.EcoGII-modified pBR322 DNA (Dam^−^) to see any enhancement of activity due to frequent adenine methylation. M.EcoGII is capable of methylating all adenines in DNA substrates except in polyA tracks (Murray et al., 2018). Ahi29725I activity was enhanced on M.EcoGII-methylated DNA substrate (Supplementary Figure S12). Three large fragments of Dam^+^ DNA were further digested into smaller fragments after M.EcoGII methylation. However, it was not clear whether the enhanced activity was due to 6mA-dependent relaxed sequence recognition (e.g., cleavage near the Cm6ATC star site or Sm6ATS sites, S = G and C). The enhanced activity on M.EcoGII-modified DNA remains to be characterized in future by using defined modified oligos or restriction digestion/NGS sequencing mapping of M.EcoGII-modified λDNA.

In digestion of methylated duplex oligos with a single G6mATC site, it was noted that Ahi29725I preferentially cleaved fully methylated oligos (M+/M+) over hemi-modified substrates (M+/M− or M−/M+). However, Apa233I endonuclease was able to cut both fully modified and hemi-modified oligos (Figure 4). This discrepancy in methylation dependence between the two enzymes is unexplained.

If the *in vitro* divalent cation requirement of Ahi29725I was relevant in cells, invading 6mA-modified DNA would be digested by Mg^2+^-bound Ahi29725I. By contrast, activation of the non-specific endonuclease activity of Ahi29725I with Mn^2+^, Co^2+^, or Ni^2+^ in the active site could lead to cleavage of both cellular and invading DNA regardless of modifications, triggering cell death and preventing phage release.

### PLD—wH endonucleases

We identified 27 predicted PLD—wH fusion endonucleases in bacterial genomes. Two putative restriction genes from *Anaerolineaceae bacterium* (Aba4572I) and *Chloroflexi bacterium* (CbaI) were cloned into the pTXB1 expression vector. However, upon IPTG induction, no over-expressed proteins were detected. In the gene neighborhood analysis, the Aba45721 ORF resides in a genomic region of (1) DNA MTase (predicted specificity CGATCG, amino-MTase), (2) PLD-wH endonuclease, and (3) and (4) hypothetical proteins. If the CGATCG site is methylated to become CGm6ATCG, it would be a substrate for the PLD—wH endonuclease, which could potentially result in self-restriction. The CbaI enzyme is located in a region with (1) Leu-tRNA ligase, (2) restriction endonuclease, (3) PLD—wH endonuclease, (4) hypothetical protein, and (5) dimethyl-menaquinone MTase. Since the transformation of Aba4572I and CbaI was less toxic in Dam^−^ cells in a non-induced condition (see Figure 3), the lack of expression in Dam^−^ cells is surprising and requires further investigation. Expression of two more PLD-wH fusion proteins containing the conserved catalytic residues HxDx(4)K and HxEx(4)K in the PLD endonuclease domain in *E. coli* Dam^−^ cells was not successful due to toxicity. More work is necessary to explain the reasons for the poor expression of PLD-wH fusion endonucleases in *E. coli*.

### NTD—wH—NTPase fusions

The N-terminal domain-wH-NTPase fusions are usually paired with another catalytic subunit, such as an McrC (Ross et al., 1989) McrC-like protein with a PD-(D/E)XK endonuclease motif (Pieper and Pingoud, 2002). This arrangement is reminiscent of McrBC, a type IV restriction system acting on modified cytosines (Stewart et al., 2000). We only made one unsuccessful attempt to purify a putative heterodimeric NTD—wH—NTPase/McrC complex. Therefore, we have not studied the possible activity of these enzymes toward 6mA-containing DNA or their more general activity in the restriction of modified DNA. More work is needed to characterize this group of putative type IV restriction systems with wH-NTPase fusion.

## Discussion

### wH domain as a sensor of fully methylated ApT in a dsDNA context

The wH domain was first associated with adenine methylation because of its presence in the C-terminal region of *E. coli* and phage T4 Dam methyltransferases (Teichmann et al., 2012), and later because of its presence in the adenine methylation-dependent DpnI restriction endonuclease. A subsequent study on DpnI showed that the wH domain binds dsDNA at fully methylated ApT sites without base flipping. The two methyl groups, which are in close proximity, are bound in a single pocket of the wH domain of DpnI (Mierzejewska et al., 2014). The study on DpnI also showed that the specificity of the domain for the flanking sequence was somewhat relaxed with respect to the Dam Gm6ATC consensus and the Sm6ATS sites (where S stands for G or C) (Siwek et al., 2012). In this study, we show that the properties of an adenine methylation reader carry over to many fusions with HNH, GIY-YIG, and likely also PLD endonuclease domains. If methylation is seen in the ApT context, there is a preference for fully methylated sites except for Apa233I (see Figures 3, 4). Our study shows that in all tested fusion proteins, the wH domain can operate as an adenine methylation reader for the Gm6ATC context. A study on the wH—GIY-YIG endonucleases further indicates that additional cleavage sites are likely created when DNA is hypermethylated by M.EcoGII (see Supplementary Figure S12). Hence, the wH domains of the wH—GIY-YIG endonucleases also suggest that the Gm6ATC preference may be relaxed, but the star binding sites remain to be characterized (star sites are usually defined as DNA sequences with one base off from the cognate site; if there are two bases off, these sequences are usually called non-cognate sites; Pingoud et al., 2016).

**Figure 10:**
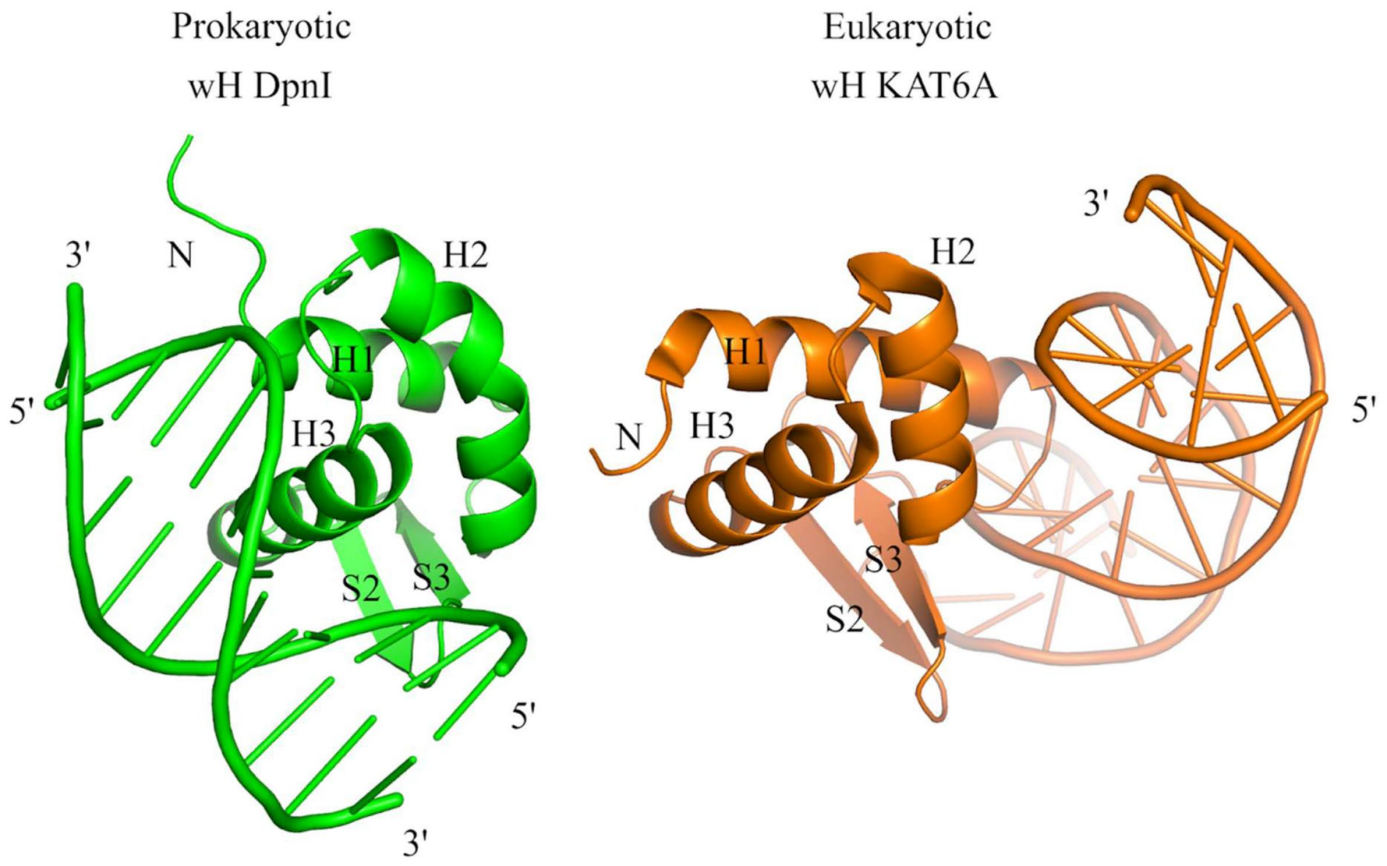
Structural comparison of the wH domains of DpnI (G6mATC) with bacterial origin and mammalian KAT6A protein that recognizes unmodified CpG sites. The docking of wH domains on dsDNA shows a major difference in recognition. The wH domain-containing protein KAT6A is a histone lysine acetyltransferase that acetylates lysine residues in histones H3 and H4 (*in vitro*) (Weber et al., 2023).

The identification of prokaryotic winged helix domains as sensors of adenine methylation contrasts with the role of some eukaryotic winged helix domains as sensors of non-methylated CpG (Stielow et al., 2021; Becht et al., 2023; Weber et al., 2023). The superposition of the winged helix domains of prokaryotic DpnI (Mierzejewska et al., 2014) and eukaryotic KAT6A (also called histone acetyltransferase KAT6A, lysine acetyltransferase 6A, zinc finger protein 220, and MYST-3) (Weber et al., 2023) shows that the dsDNA molecules are bound to opposite faces of the wH domain (Figure 10), indicating that the two DNA-binding modes have likely evolved independently for needs that are characteristic for prokaryotes (sensing of Dam methylation) and eukaryotes (sensing of the absence of CpG methylation).

### Cooperation with endonuclease domains

For most NTP-independent MDREs, there is a clear division of labor between the modification reader and endonuclease domains. The former recruits the enzyme to modification sites, and the latter cleaves the DNA at a distance from the recognition site, which is likely defined by the length of the linker that connects the two domains. The nuclease domain has generally low or only a very relaxed sequence specificity, and it is likely not modification-specific. How the modification sensor domain keeps the activity of the nuclease domain in check is not well understood. In some cases, it can be shown that the linker has an inhibitory role for the endonuclease that is only relieved once modified DNA is bound to the reader domain and the complex reorganizes structurally (Pastor et al., 2021). The PUA—wH—HNH (HhiV4I) and wH—GIY-YIG (Ahi29725I and Apa233I) that were tested by run-off sequencing are consistent with this expectation. As for the cytosine modification-specific MDREs, cleavage occurred mostly at a distance from the recognition sequence except for the BisI family REases (e.g., Eco15I and NhoI) that cut within the recognition sequence GCNGC with 2–4 modified cytosines (Xu et al., 2016).

Among the wH fusion endonucleases, DpnI and its isochizomers are the exception to the rule that cleavage occurs always outside of and not within the recognition sequence. Mechanically, DpnI DNA cleavage within the recognition sequence is a consequence of the fact that the endonuclease domain has separate sequence and modification specificities (Siwek et al., 2012). In this scenario, the role of the wH domain is similar to the role of the extra specificity domain in type IIE restriction endonucleases (Roberts et al., 2003), except that the target sequence contains a modified base. Type IIE restriction endonucleases are one of the subfamilies that require a pair of target sequences in order to show activity (Senesac and Allen, 1995; Colandene and Topal, 1998). Run-off sequencing shows that FcyTI and Psp4BI cleave inside the recognition sequence, like DpnI, pointing to the separate sequence and modification specificity of the catalytic PD-(D/E)XK domain. Given the high sequence conservation of the PD-(D/E)XK—wH endonuclease family, it is likely that this property is general for the entire family.

### Sensors/readers for N6-methyladenine in DNA

Despite the many roles of DNA adenine methylation in prokaryotes (and eukaryotic organelles), the repertoire of reader domains for m6A in DNA is still surprisingly limited (Iyer et al., 2016). Perhaps the best-known adenine methylation sensors are the YTH (Liao et al., 2018) domains, which belong to (or are related to) the PUA superfamily domains. The PUA superfamily domains are believed to flip the modified 2′-deoxynucleotide out of duplex DNA (Shao et al., 2014) or to bind a single nucleotide in RNA in the reader pocket (Li et al., 2014; Xu et al., 2014). Therefore, at least when acting in isolation, they can be considered as sensors of a single modified adenine. Consistent with this role, most YTH domains sense adenine methylation in RNA rather than DNA. However, some YTH and ASCH domains in prokaryotes are considered as DNA adenine methylation sensors (Iyer et al., 2016). For the ASCH domains, this remains to be experimentally shown, since currently only a 4mC reader role is experimentally supported (Stanislauskiene et al., 2020). Apart from the YTH and ASCH domains, the HARE-HTH and RAMA (Restriction enzyme and Adenine Methylation Associated) domains have also been suggested to serve as readers of adenine methylation in DNA (Fomenkov et al., 1994; Teichmann et al., 2012). The HARE-HTH domains are related to winged helix domains, which would be consistent with a role as adenine methylation sensors. However, they have an extra helix inserted into the HTH motif of the winged helix domain, and recent analysis suggests that they are more likely to sense cytosine modifications (Aravind and Iyer, 2012). Unlike the HARE-HTH domains, the RAMA domains are unrelated to the wH domain in fold (Yang et al., 2023). For the RAMA domain-containing MPND protein, there is some biochemical evidence for adenine methylation sensing (Kweon et al., 2019). However, a preference for adenine-methylated DNA could not be experimentally confirmed (Yang et al., 2023). We noticed the occurrence of RAMA—Mrr catalytic domain (PD-QXK)—NTPase (three-domain fusion) and GIY-YIG—RAMA (two-domain fusion) in prokaryotes, which might indicate that the RAMA domain is utilized similarly to the wH domain in these fusions. Future studies will be focused on the characterization of YTH-NTPase, YTH-HNH, and RAMA fusion endonucleases as 6mA readers/sensors in type IV restriction systems.

## Data availability statement

The original contributions presented in the study are included in the article/Supplementary material, further inquiries can be directed to the corresponding author.

## Author contributions

IH: Data curation, Formal analysis, Investigation, Methodology, Validation, Writing – review & editing. DH: Data curation, Formal analysis, Investigation, Methodology, Writing – review & editing. WY: Data curation, Formal analysis, Investigation, Methodology, Software, Writing – review & editing. TV: Data curation, Investigation, Writing – review & editing. AH: Data curation, Investigation, Writing – review & editing. TL: Data curation, Formal analysis, Investigation, Methodology, Validation, Writing – review & editing. LE: Data curation, Formal analysis, Investigation, Methodology, Software, Writing – review & editing. MB: Conceptualization, Formal analysis, Funding acquisition, Investigation, Methodology, Resources, Supervision, Visualization, Writing – original draft, Writing – review & editing. S-yX: Conceptualization, Formal analysis, Investigation, Methodology, Resources, Supervision, Validation, Writing – original draft, Writing – review & editing.

## Footnotes

^1^ https://itol.embl.de/

## References

[ref1] Aravind L. Iyer L. M. (2012). The HARE-HTH and associated domains: novel modules in the coordination of epigenetic DNA and protein modifications. Cell Cycle 11, 119–131. doi: 10.4161/cc.11.1.18475, PMID: 22186017 PMC3272235

[ref2] Au K. G. Welsh K. Modrich P. (1992). Initiation of methyl-directed mismatch repair. J. Biol. Chem. 267, 12142–12148. doi: 10.1016/S0021-9258(19)49816-5 1601880

[ref3] Becht D. C. Klein B. J. Kanai A. Jang S. M. Cox K. L. Zhou B. R.. (2023). MORF and MOZ acetyltransferases target unmethylated CpG islands through the winged helix domain. Nat. Commun. 14:697. doi: 10.1038/s41467-023-36368-5, PMID: 36754959 PMC9908889

[ref4] Bertonati C. Punta M. Fischer M. Yachdav G. Forouhar F. Zhou W.. (2009). Structural genomics reveals EVE as a new ASCH/PUA-related domain. Proteins 75, 760–773. doi: 10.1002/prot.22287, PMID: 19191354 PMC4080787

[ref5] Borgaro J. G. Zhu Z. (2013). Characterization of the 5-hydroxymethylcytosine-specific DNA restriction endonucleases. Nucleic Acids Res. 41, 4198–4206. doi: 10.1093/nar/gkt102, PMID: 23482393 PMC3627594

[ref6] Boye E. Lobner-Olesen A. (1990). The role of dam methyltransferase in the control of DNA replication in *E. coli*. Cell 62, 981–989. doi: 10.1016/0092-8674(90)90272-G, PMID: 2203541

[ref7] Brennan R. G. (1993). The winged-helix DNA-binding motif: another helix-turn-helix takeoff. Cell 74, 773–776. doi: 10.1016/0092-8674(93)90456-Z, PMID: 8374950

[ref8] Bujnicki J. M. Rychlewski L. (2001). Grouping together highly diverged PD-(D/E)XK nucleases and identification of novel superfamily members using structure-guided alignment of sequence profiles. J. Mol. Microbiol. Biotechnol. 3, 69–72. doi: 10.1016/s0378-1119(01)00405-x PMID: 11200231

[ref9] Cerrudo C. S. Ghiringhelli P. D. Gomez D. E. (2014). Protein universe containing a PUA RNA-binding domain. FEBS J. 281, 74–87. doi: 10.1111/febs.12602, PMID: 24393395

[ref10] Chan S. H. Bao Y. Ciszak E. Laget S. Xu S. Y. (2007). Catalytic domain of restriction endonuclease BmrI as a cleavage module for engineering endonucleases with novel substrate specificities. Nucleic Acids Res. 35, 6238–6248. doi: 10.1093/nar/gkm665, PMID: 17855396 PMC2094064

[ref11] Cohen-Karni D. Xu D. Apone L. Fomenkov A. Sun Z. Davis P. J.. (2011). The MspJI family of modification-dependent restriction endonucleases for epigenetic studies. Proc. Natl. Acad. Sci. USA 108, 11040–11045. doi: 10.1073/pnas.1018448108, PMID: 21690366 PMC3131316

[ref12] Colandene J. D. Topal M. D. (1998). The domain organization of NaeI endonuclease: separation of binding and catalysis. Proc. Natl. Acad. Sci. USA 95, 3531–3536. doi: 10.1073/pnas.95.7.3531, PMID: 9520400 PMC19870

[ref13] Czapinska H. Kowalska M. Zagorskaite E. Manakova E. Slyvka A. Xu S. Y.. (2018). Activity and structure of EcoKMcrA. Nucleic Acids Res. 46, 9829–9841. doi: 10.1093/nar/gky731, PMID: 30107581 PMC6182155

[ref14] Dunin-Horkawicz S. Feder M. Bujnicki J. M. (2006). Phylogenomic analysis of the GIY-YIG nuclease superfamily. BMC Genomics 7:98. doi: 10.1186/1471-2164-7-98, PMID: 16646971 PMC1564403

[ref15] Flick K. E. Jurica M. S. Monnat R. J. Jr. Stoddard B. L. (1998). DNA binding and cleavage by the nuclear intron-encoded homing endonuclease I-PpoI. Nature 394, 96–101. doi: 10.1038/27952, PMID: 9665136

[ref16] Fomenkov A. Xiao J. P. Dila D. Raleigh E. Xu S. Y. (1994). The 'endo-blue method' for direct cloning of restriction endonuclease genes in *E. coli*. Nucleic Acids Res. 22, 2399–2403. doi: 10.1093/nar/22.12.2399, PMID: 8036170 PMC523701

[ref17] Frickey T. Lupas A. (2004). CLANS: a Java application for visualizing protein families based on pairwise similarity. Bioinformatics 20, 3702–3704. doi: 10.1093/bioinformatics/bth444, PMID: 15284097

[ref18] Gajiwala K. S. Burley S. K. (2000). Winged helix proteins. Curr. Opin. Struct. Biol. 10, 110–116. doi: 10.1016/S0959-440X(99)00057-3 10679470

[ref19] Gajiwala K. S. Chen H. Cornille F. Roques B. P. Reith W. Mach B.. (2000). Structure of the winged-helix protein hRFX1 reveals a new mode of DNA binding. Nature 403, 916–921. doi: 10.1038/35002634, PMID: 10706293

[ref20] Grazulis S. Manakova E. Roessle M. Bochtler M. Tamulaitiene G. Huber R.. (2005). Structure of the metal-independent restriction enzyme BfiI reveals fusion of a specific DNA-binding domain with a nonspecific nuclease. Proc. Natl. Acad. Sci. U. S. A. 102, 15797–15802. doi: 10.1073/pnas.0507949102, PMID: 16247004 PMC1266039

[ref21] Heitman J. Model P. (1990). Substrate recognition by the EcoRI endonuclease. Proteins 7, 185–197. doi: 10.1002/prot.340070207 2139225

[ref22] Horton J. R. Wang H. Mabuchi M. Y. Zhang X. Roberts R. J. Zheng Y.. (2014). Modification-dependent restriction endonuclease, MspJI, flips 5-methylcytosine out of the DNA helix. Nucleic Acids Res. 42, 12092–12101. doi: 10.1093/nar/gku871, PMID: 25262349 PMC4231741

[ref23] Hosford C. J. Bui A. Q. Chappie J. S. (2020). The structure of the *Thermococcus gammatolerans* McrB N-terminal domain reveals a new mode of substrate recognition and specificity among McrB homologs. J. Biol. Chem. 295, 743–756. doi: 10.1016/S0021-9258(17)49932-7, PMID: 31822563 PMC6970917

[ref24] Iyer L. M. Burroughs A. M. Aravind L. (2006). The ASCH superfamily: novel domains with a fold related to the PUA domain and a potential role in RNA metabolism. Bioinformatics 22, 257–263. doi: 10.1093/bioinformatics/bti767, PMID: 16322048

[ref25] Iyer L. M. Zhang D. Aravind L. (2016). Adenine methylation in eukaryotes: apprehending the complex evolutionary history and functional potential of an epigenetic modification. Bioessays 38, 27–40. doi: 10.1002/bies.201500104, PMID: 26660621 PMC4738411

[ref26] Jablonska J. Matelska D. Steczkiewicz K. Ginalski K. (2017). Systematic classification of the his-me finger superfamily. Nucleic Acids Res. 45, 11479–11494. doi: 10.1093/nar/gkx924, PMID: 29040665 PMC5714182

[ref27] Janosi L. Yonemitsu H. Hong H. Kaji A. (1994). Molecular cloning and expression of a novel hydroxymethylcytosine-specific restriction enzyme (PvuRts1I) modulated by glucosylation of DNA. J. Mol. Biol. 242, 45–61. doi: 10.1006/jmbi.1994.1556, PMID: 8078071

[ref28] Josephs E. A. Zheng T. Marszalek P. E. (2015). Atomic force microscopy captures the initiation of methyl-directed DNA mismatch repair. DNA Repair 35, 71–84. doi: 10.1016/j.dnarep.2015.08.006, PMID: 26466357 PMC4651853

[ref29] Kabsch W. Sander C. (1983). Dictionary of protein secondary structure: pattern recognition of hydrogen-bonded and geometrical features. Biopolymers 22, 2577–2637. doi: 10.1002/bip.360221211, PMID: 6667333

[ref30] Kaminska K. H. Kawai M. Boniecki M. Kobayashi I. Bujnicki J. M. (2008). Type II restriction endonuclease R.Hpy188I belongs to the GIY-YIG nuclease superfamily, but exhibits an unusual active site. BMC Struct. Biol. 8:48. doi: 10.1186/1472-6807-8-48, PMID: 19014591 PMC2630997

[ref31] Kazrani A. A. Kowalska M. Czapinska H. Bochtler M. (2014). Crystal structure of the 5hmC specific endonuclease PvuRts1I. Nucleic Acids Res. 42, 5929–5936. doi: 10.1093/nar/gku186, PMID: 24634440 PMC4027163

[ref32] Kosinski J. Feder M. Bujnicki J. M. (2005). The PD-(D/E)XK superfamily revisited: identification of new members among proteins involved in DNA metabolism and functional predictions for domains of (hitherto) unknown function. BMC Bioinformatics 6:172. doi: 10.1186/1471-2105-6-172, PMID: 16011798 PMC1189080

[ref33] Kweon S. M. Chen Y. Moon E. Kvederaviciute K. Klimasauskas S. Feldman D. E. (2019). An adversarial DNA N(6)-Methyladenine-sensor network preserves Polycomb silencing. Mol. Cell 74, 1138–1147.e6. doi: 10.1016/j.molcel.2019.03.018, PMID: 30982744 PMC6591016

[ref34] Lai E. Clark K. L. Burley S. K. Darnell J. E. Jr. (1993). Hepatocyte nuclear factor 3/fork head or "winged helix" proteins: a family of transcription factors of diverse biologic function. Proc. Natl. Acad. Sci. U. S. A. 90, 10421–10423. doi: 10.1073/pnas.90.22.10421, PMID: 8248124 PMC47788

[ref35] Li F. Zhao D. Wu J. Shi Y. (2014). Structure of the YTH domain of human YTHDF2 in complex with an m(6)a mononucleotide reveals an aromatic cage for m(6)a recognition. Cell Res. 24, 1490–1492. doi: 10.1038/cr.2014.153, PMID: 25412658 PMC4260351

[ref36] Liao S. Sun H. Xu C. Domain Y. T. H. (2018). A family of N(6)-methyladenosine (m(6)a) readers. Genomics Proteomics Bioinformatics 16, 99–107. doi: 10.1016/j.gpb.2018.04.002, PMID: 29715522 PMC6112328

[ref37] Liu G. Ou H. Y. Wang T. Li L. Tan H. Zhou X.. (2010). Cleavage of phosphorothioated DNA and methylated DNA by the type IV restriction endonuclease ScoMcrA. PLoS Genet. 6:e1001253. doi: 10.1371/journal.pgen.1001253, PMID: 21203499 PMC3009677

[ref38] Lu X. Huang F. Cheng R. Zhu B. (2023). A unique m6A-dependent restriction endonuclease from an archaeal virus. Microbiol. Spectr. 11:e0426222. doi: 10.1128/spectrum.04262-22, PMID: 36946751 PMC10101028

[ref39] Lutz T. Flodman K. Copelas A. Czapinska H. Mabuchi M. Fomenkov A.. (2019). A protein architecture guided screen for modification dependent restriction endonucleases. Nucleic Acids Res. 47, 9761–9776. doi: 10.1093/nar/gkz755, PMID: 31504772 PMC6765204

[ref40] Makarova K. S. Wolf Y. I. Snir S. Koonin E. V. (2011). Defense islands in bacterial and archaeal genomes and prediction of novel defense systems. J. Bacteriol. 193, 6039–6056. doi: 10.1128/JB.05535-11 21908672 PMC3194920

[ref41] Marinus M. G. Casadesus J. (2009). Roles of DNA adenine methylation in host-pathogen interactions: mismatch repair, transcriptional regulation, and more. FEMS Microbiol. Rev. 33, 488–503. doi: 10.1111/j.1574-6976.2008.00159.x, PMID: 19175412 PMC2941194

[ref42] Mehta P. Katta K. Krishnaswamy S. (2004). HNH family subclassification leads to identification of commonality in the his-me endonuclease superfamily. Protein Sci. 13, 295–300. doi: 10.1110/ps.03115604, PMID: 14691243 PMC2286527

[ref43] Mierzejewska K. Siwek W. Czapinska H. Kaus-Drobek M. Radlinska M. Skowronek K.. (2014). Structural basis of the methylation specificity of R.DpnI. Nucleic Acids Res. 42, 8745–8754. doi: 10.1093/nar/gku546, PMID: 24966351 PMC4117772

[ref44] Miyazono K. Furuta Y. Watanabe-Matsui M. Miyakawa T. Ito T. Kobayashi I.. (2014). A sequence-specific DNA glycosylase mediates restriction-modification in Pyrococcus abyssi. Nat. Commun. 5:3178. doi: 10.1038/ncomms4178, PMID: 24458096

[ref45] Murray I. A. Morgan R. D. Luyten Y. Fomenkov A. Correa I. R. Jr. Dai N.. (2018). The non-specific adenine DNA methyltransferase M.EcoGII. Nucleic Acids Res. 46, 840–848. doi: 10.1093/nar/gkx1191, PMID: 29228259 PMC5778455

[ref46] Orlowski J. Bujnicki J. M. (2008). Structural and evolutionary classification of type II restriction enzymes based on theoretical and experimental analyses. Nucleic Acids Res. 36, 3552–3569. doi: 10.1093/nar/gkn175, PMID: 18456708 PMC2441816

[ref47] Pastor M. Czapinska H. Helbrecht I. Krakowska K. Lutz T. Xu S. Y.. (2021). Crystal structures of the EVE-HNH endonuclease VcaM4I in the presence and absence of DNA. Nucleic Acids Res. 49, 1708–1723. doi: 10.1093/nar/gkaa1218, PMID: 33450012 PMC7897488

[ref48] Pieper U. Pingoud A. (2002). A mutational analysis of the PD…D/EXK motif suggests that McrC harbors the catalytic center for DNA cleavage by the GTP-dependent restriction enzyme McrBC from *Escherichia coli*. Biochemistry 41, 5236–5244. doi: 10.1021/bi0156862, PMID: 11955073

[ref49] Pingoud A. Fuxreiter M. Pingoud V. Wende W. (2005). Type II restriction endonucleases: structure and mechanism. Cell. Mol. Life Sci. 62, 685–707. doi: 10.1007/s00018-004-4513-1 15770420 PMC11924531

[ref50] Pingoud A. Jeltsch A. (2001). Structure and function of type II restriction endonucleases. Nucleic Acids Res. 29, 3705–3727. doi: 10.1093/nar/29.18.3705, PMID: 11557805 PMC55916

[ref51] Pingoud A. Wilson G. G. Wende W. (2016). Type II restriction endonucleases - a historical perspective and more. Nucleic Acids Res. 44:8011. doi: 10.1093/nar/gkw513, PMID: 27270082 PMC5027491

[ref52] Pommer A. J. Cal S. Keeble A. H. Walker D. Evans S. J. Kuhlmann U. C.. (2001). Mechanism and cleavage specificity of the H-N-H endonuclease colicin E9. J. Mol. Biol. 314, 735–749. doi: 10.1006/jmbi.2001.5189 11733993

[ref53] Roberts R. J. Belfort M. Bestor T. Bhagwat A. S. Bickle T. A. Bitinaite J.. (2003). A nomenclature for restriction enzymes, DNA methyltransferases, homing endonucleases and their genes. Nucleic Acids Res. 31, 1805–1812. doi: 10.1093/nar/gkg274, PMID: 12654995 PMC152790

[ref54] Roberts R. J. Cheng X. (1998). Base flipping. Annu. Rev. Biochem. 67, 181–198. doi: 10.1146/annurev.biochem.67.1.181 9759487

[ref55] Ross T. K. Achberger E. C. Braymer H. D. (1989). Identification of a second polypeptide required for McrB restriction of 5-methylcytosine-containing DNA in *Escherichia coli* K12. Mol. Gen. Genet. 216, 402–407. doi: 10.1007/BF00334382, PMID: 2664457

[ref56] Sasnauskas G. Zakrys L. Zaremba M. Cosstick R. Gaynor J. W. Halford S. E.. (2010). A novel mechanism for the scission of double-stranded DNA: BfiI cuts both 3′-5′ and 5′-3′ strands by rotating a single active site. Nucleic Acids Res. 38, 2399–2410. doi: 10.1093/nar/gkp1194, PMID: 20047964 PMC2853115

[ref57] Schwartz T. Behlke J. Lowenhaupt K. Heinemann U. Rich A. (2001). Structure of the DLM-1-Z-DNA complex reveals a conserved family of Z-DNA-binding proteins. Nat. Struct. Biol. 8, 761–765. doi: 10.1038/nsb0901-761 11524677

[ref58] Senesac J. H. Allen J. R. (1995). Oligonucleotide activation of the type IIe restriction enzyme NaeI for digestion of refractory sites. BioTechniques 19, 990–993. PMID: 8747667

[ref59] Shao C. Wang C. Zang J. (2014). Structural basis for the substrate selectivity of PvuRts1I, a 5-hydroxymethylcytosine DNA restriction endonuclease. Acta Crystallogr. D Biol. Crystallogr. 70, 2477–2486. doi: 10.1107/S139900471401606X, PMID: 25195760 PMC4157451

[ref60] Siwek W. Czapinska H. Bochtler M. Bujnicki J. M. Skowronek K. (2012). Crystal structure and mechanism of action of the N6-methyladenine-dependent type IIM restriction endonuclease R.DpnI. Nucleic Acids Res. 40, 7563–7572. doi: 10.1093/nar/gks428, PMID: 22610857 PMC3424567

[ref61] Slyvka A. Zagorskaite E. Czapinska H. Sasnauskas G. Bochtler M. (2019). Crystal structure of the EcoKMcrA N-terminal domain (NEco): recognition of modified cytosine bases without flipping. Nucleic Acids Res. 47, 11943–11955. doi: 10.1093/nar/gkz1017, PMID: 31724709 PMC7145662

[ref62] Sokolowska M. Czapinska H. Bochtler M. (2009). Crystal structure of the beta beta alpha-me type II restriction endonuclease Hpy99I with target DNA. Nucleic Acids Res. 37, 3799–3810. doi: 10.1093/nar/gkp228, PMID: 19380375 PMC2699513

[ref63] Sokolowska M. Czapinska H. Bochtler M. (2011). Hpy188I-DNA pre- and post-cleavage complexes--snapshots of the GIY-YIG nuclease mediated catalysis. Nucleic Acids Res. 39, 1554–1564. doi: 10.1093/nar/gkq821, PMID: 20935048 PMC3045582

[ref64] Stanislauskiene R. Laurynenas A. Rutkiene R. Aucynaite A. Tauraite D. Meskiene R.. (2020). YqfB protein from *Escherichia coli*: an atypical amidohydrolase active towards N(4)-acylcytosine derivatives. Sci. Rep. 10:788. doi: 10.1038/s41598-020-57664-w, PMID: 31964920 PMC6972931

[ref65] Stewart F. J. Panne D. Bickle T. A. Raleigh E. A. (2000). Methyl-specific DNA binding by McrBC, a modification-dependent restriction enzyme. J. Mol. Biol. 298, 611–622. doi: 10.1006/jmbi.2000.3697, PMID: 10788324

[ref66] Stielow B. Zhou Y. Cao Y. Simon C. Pogoda H. M. Jiang J.. (2021). The SAM domain-containing protein 1 (SAMD1) acts as a repressive chromatin regulator at unmethylated CpG islands. Sci. Adv. 7:eabf2229. doi: 10.1126/sciadv.abf2229, PMID: 33980486 PMC8115922

[ref67] Sukackaite R. Grazulis S. Tamulaitis G. Siksnys V. (2012). The recognition domain of the methyl-specific endonuclease McrBC flips out 5-methylcytosine. Nucleic Acids Res. 40, 7552–7562. doi: 10.1093/nar/gks332, PMID: 22570415 PMC3424535

[ref68] Tang Q. Rigby R. E. Young G. R. Hvidt A. K. Davis T. Tan T. K.. (2021). Adenosine-to-inosine editing of endogenous Z-form RNA by the deaminase ADAR1 prevents spontaneous MAVS-dependent type I interferon responses. Immunity 54, 1961–1975.e5. doi: 10.1016/j.immuni.2021.08.011, PMID: 34525337 PMC8459395

[ref69] Teichmann M. Dumay-Odelot H. Fribourg S. (2012). Structural and functional aspects of winged-helix domains at the core of transcription initiation complexes. Transcription 3, 2–7. doi: 10.4161/trns.3.1.18917, PMID: 22456313

[ref70] Wah D. A. Hirsch J. A. Dorner L. F. Schildkraut I. Aggarwal A. K. (1997). Structure of the multimodular endonuclease FokI bound to DNA. Nature 388, 97–100. doi: 10.1038/40446, PMID: 9214510

[ref71] Weber L. M. Jia Y. Stielow B. Gisselbrecht S. S. Cao Y. Ren Y.. (2023). The histone acetyltransferase KAT6A is recruited to unmethylated CpG islands via a DNA binding winged helix domain. Nucleic Acids Res. 51, 574–594. doi: 10.1093/nar/gkac1188, PMID: 36537216 PMC9881136

[ref72] Wolberger C. Campbell R. (2000). New perch for the winged helix. Nat. Struct. Biol. 7, 261–262. doi: 10.1038/74004 10742162

[ref73] Wu C. C. Lin J. L. J. Yuan H. S. (2020). Structures, mechanisms, and functions of his-me finger nucleases. Trends Biochem. Sci. 45, 935–946. doi: 10.1016/j.tibs.2020.07.002, PMID: 32807610

[ref74] Xu S. Y. Klein P. Degtyarev S. Roberts R. J. (2016). Expression and purification of the modification-dependent restriction enzyme BisI and its homologous enzymes. Sci. Rep. 6:28579. doi: 10.1038/srep28579, PMID: 27353146 PMC4926085

[ref75] Xu D. Shao J. Song H. Wang J. (2020). The YTH Domain family of N6-Methyladenosine “readers” in the diagnosis and prognosis of colonic adenocarcinoma. Biomed. Res. Int. 2020:9502560. doi: 10.1155/2020/9502560 32596399 PMC7277069

[ref76] Xu C. Wang X. Liu K. Roundtree I. A. Tempel W. Li Y.. (2014). Structural basis for selective binding of m6A RNA by the YTHDC1 YTH domain. Nat. Chem. Biol. 10, 927–929. doi: 10.1038/nchembio.1654, PMID: 25242552

[ref77] Yang M. Li X. Tian Z. Ma L. Ma J. Liu Y.. Structures of MPND reveal the molecular recognition of nucleosomes. Int. J. Mol. Sci. 24:3368. doi: 10.3390/ijms24043368 PMC9963953 36834777

