# Supplementary material for: Characterization of winged helix domain fusion endonucleases as N6-methyladenine-dependent type IV restriction systems
Source: Front Microbiol. 2024 Apr 9;15:1286822. doi: 10.3389/fmicb.2024.1286822 (PMC11037411; doi:10.3389/fmicb.2024.1286822)
Supplement: Supplementary file 1 [file Data_Sheet_1.docx]

**Supplementary Materials**

**Suppl. Tables S1-S3.**

**Suppl. Figures S1-S12.**

**Raw data of CE assays on modified, hemi-modified and unmodified duplex oligos.**

**Raw data of proteomic analysis of purified enzymes**

**Characterization of winged helix domain fusion endonucleases as N6-methyladenine-dependent Type IV restriction systems**

Igor Helbrecht^1,2#^, Daniel Heiter^1#^, Weiwei Yang^1^, Tamas Vincze^1^, Andrew Hanneman^1^, Thomas Lutz^1^, Laurence Ettwiller^1^, Matthias Bochtler^2^*, Shuang-yong Xu^1^*

1. New England Biolabs, Inc. 240 County Road, Ipswich, MA 01938.

2. Institute of Biochemistry and Biophysics, Polish Academy of Sciences, Pawinskiego 5a, 02-106 Warsaw, Poland.

# These authors contributed to this work equally.

*Corresponding authors

Dr. Matthias Bochtler: [mbochtler@iimcb.gov.pl](mailto:mbochtler@iimcb.gov.pl)

and

Dr. Shuang-yong Xu: xus@neb.com

**Suppl. Table S1. Bacterial genomes with wH fusion endonucleases and predicted Dam methylases. 20 out of 1141 bacterial genomes contain both wH fusion endonucleases and predicted Dam methylase (1.75%).**

**Suppl. Table S2. Bacterial genomes with wH fusion endonucleases and M.EcoGII-like frequent adenine methylases. 8 out of 1141 bacterial genomes contain both types of enzymes (0.7%).**

**Suppl. Table S3. Bacterial genomes with wH fusion endonucleases and overlapping GATC adenine methylases. The methylase specificity prediction was based on known specificity in REBASE. 46 out of 1141 genomes contain both types of enzymes (4.03%).**

**Supplement Figures**

**Suppl. Fig. S1. Phylogenetic analysis of wH domain sequences.** wH domain sequences from the five wH-containing groups were aligned and used for construction of a phylogenetic tree. Each wH domain sequence was colored according to its group of wH domain-containing enzymes: red, PD-(D/E)XK**—**wH; pink, PLD**—**wH; yellow, wH**—**GIY-YIG; light blue, wH**—**HNH; blue, PUA**—**wH**—**HNH; green, NTD**—**wH**—**NTPase. A group of wH sequences which are homologs of both PD-(D/E)XK**—**wH and wH**—**GIY-YIG were colored in orange. The five representative wH domain sequences were highlighted in bold. Numbers at branching points are bootstrap values in %.


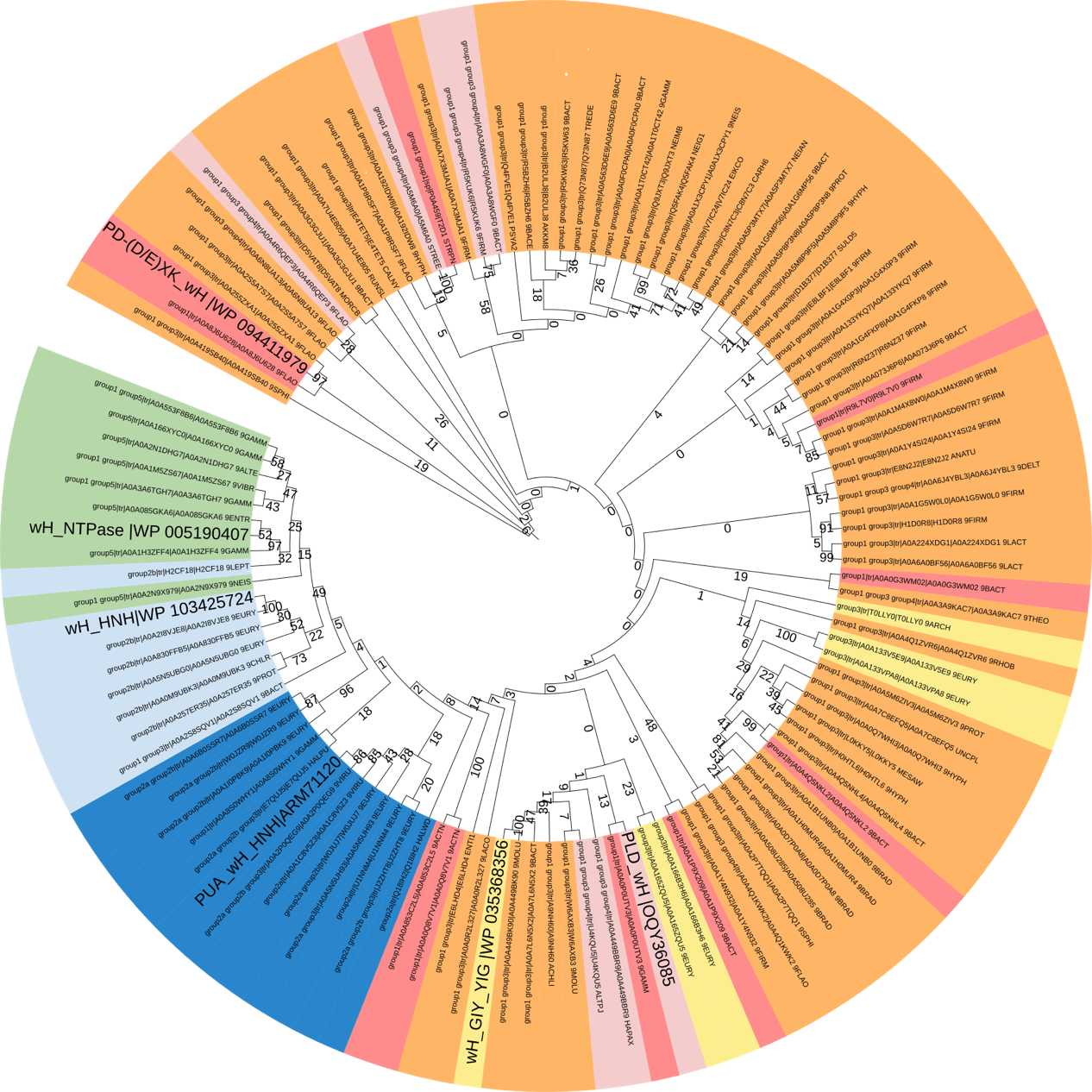


**Suppl. Fig. S2.** FcyTI and Psp4BI digestion of pBR322 (Dam^+^), pUC19 (Dam^+^ or Dam^-^), and phage λ DNA (Dam^+^ or Dam^-^). **A.** FcyTI and Psp4BI endonucleases were partially purified by affinity chromatography through chitin columns and DTT cleavage. 2-log, DNA size markers in 0.1 to 10 kb (NEB). Right: computer generated DpnI digestion pattern of pBR322 (Dam^+^) in 1% agarose gel by NEBcutter. **B.** FcyTI digestion of HindIII-linearized pUC19 (Dam^+^ or Dam^-^), and phage λ DNA (Dam^+^ or Dam^-^) (2 μg and 0.2 μg of FcyTI have equivalent of 60 and 6 U of the enzyme). MboI and DpnI were used as controls (MboI digests unmodified DNA, DpnI cuts only modified G6mATC sites). Dam^+^ λ DNA was partially modified by the host Dam methylase.


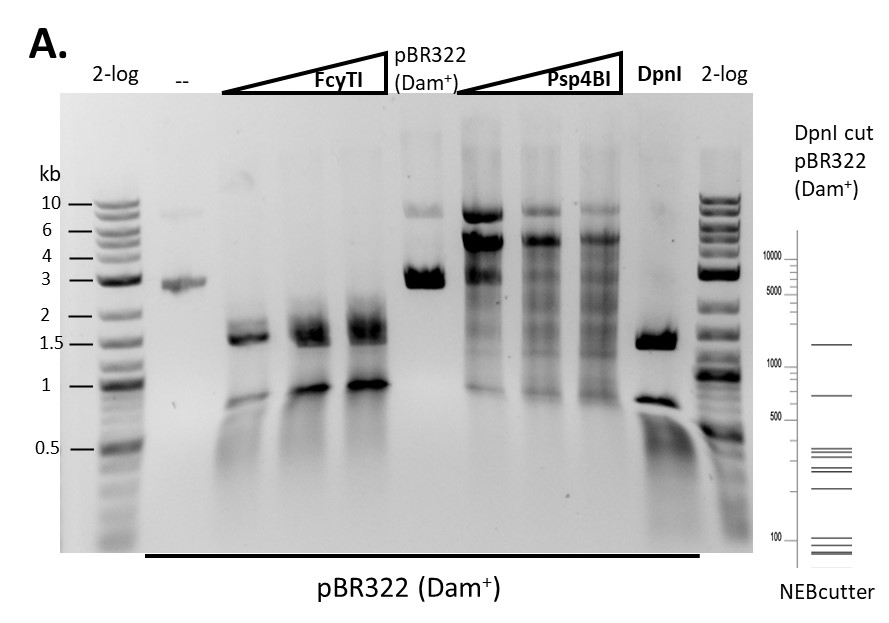


**
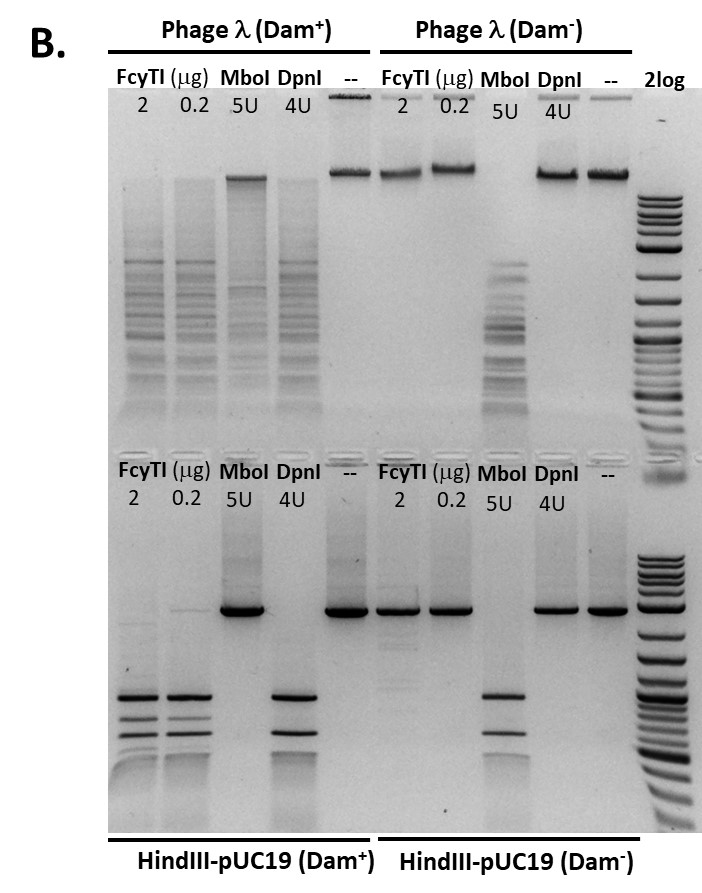
**

**Suppl. Fig. S3.** Heat inactivation of FcyTI at 65^o^C for 10-30 min. Diluted enzyme (25 to 100 U) in the restriction buffer was heated at 65^o^C for 10-30 min, and pBR322 (Dam^+^, 0.5 μg) was added and incubated at 37^o^C for 1 h. Residual activity remained after 20 min of heat treatment. FcyTI endonuclease was inactivated by heating at 65^o^C for 30 min.


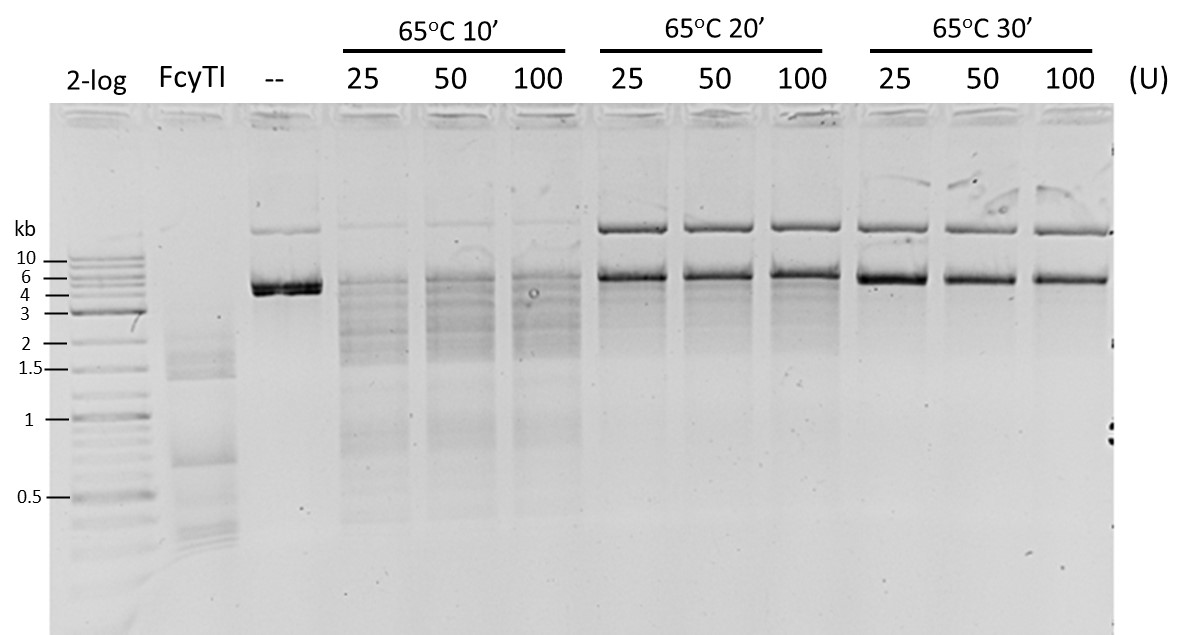


**Suppl. Fig. S4.** DNA Run-off sequencing of FcyTI and Psp4BI digested pBR322 (Dam^+^). The down and up arrows indicate the cleavage positions within the G6mA/TC sites, which is identical to that of DpnI. Uncut pBR322 was used as a control in sequencing with continuous peaks. An extra A (or T in the complement strand) was added by the terminal nucleotide transferase activity of the Taq DNA polymerase when it encountered broken templates following cleavage or nicking (1).


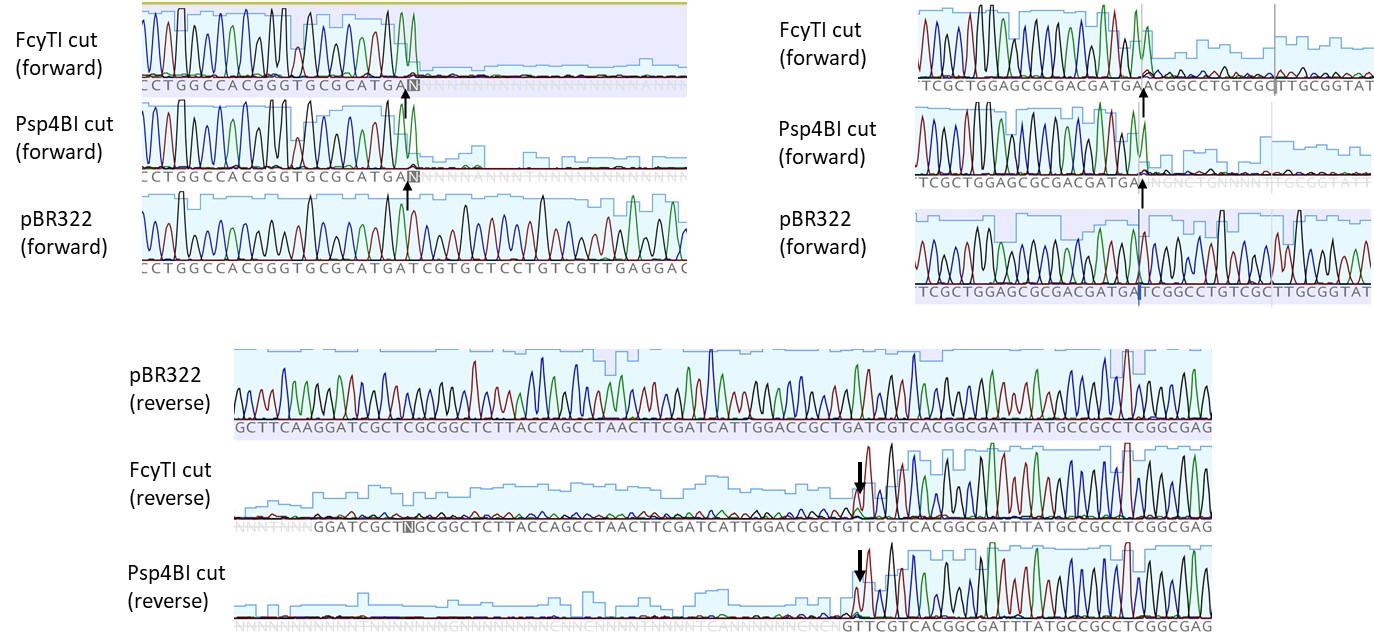


**Suppl. Fig. S5. SDS-PAGE analysis of the partially purified wH fusion endonucleases.** FcyTI, a DpnI homolog (PD-D/EXK-wH fusion); Ahi29725I and Apa233I, GIY-YIG-wH fusions; HhiV4I (PUA-wH-HNH fusion); *DpnI with BSA (200 U, R0176L, predicted MW, 29.8 kDa, NEB); *MboI with BSA (50 U, R0147L, predicted MW, 32.3 kDa, NEB); M, long range protein molecular mass marker. Purification of PLD-wH fusion endonucleases from *E. coli* lysates was not successful.

**Suppl. Fig. S6.** **DNA run-off sequencing to determine cleavage sites of HhiV4I.** Following 1 h restriction, the cleavage products were sequenced using pBR322 primers. Down arrows indicate that the top strand (as shown) was cleaved and the up arrows indicate the bottom (complementary) strand was nicked. The doublet peaks (extra A or extra T peaks) were introduced by the sequencing Taq DNA polymerase which added an A by its terminal nucleotide transferase activity when it reaches to the end of a broken template. The sudden drop of sequencing peak height also indicates the position of a broken template. Single nt overhang is found for some HNH restriction endonucleases.


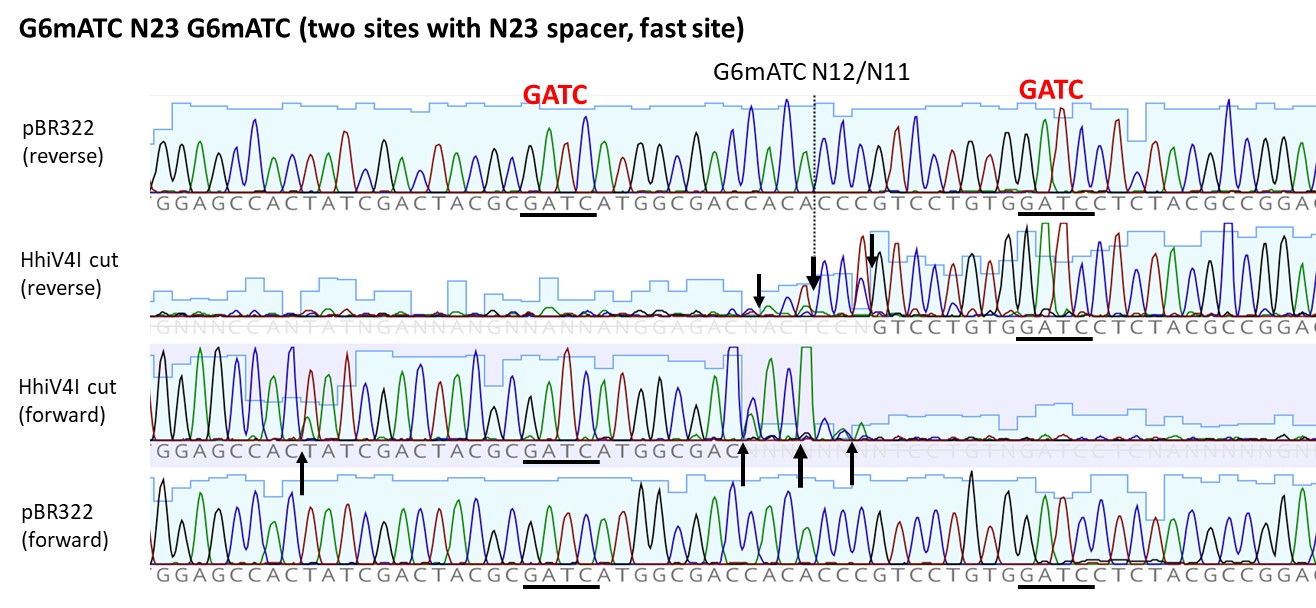


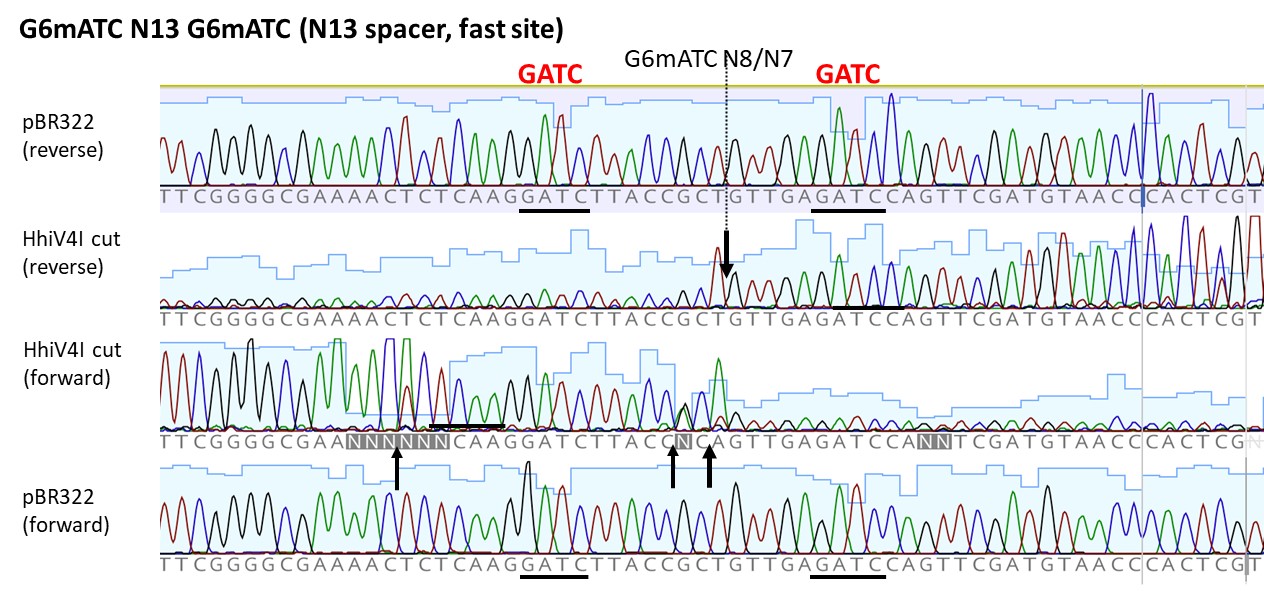


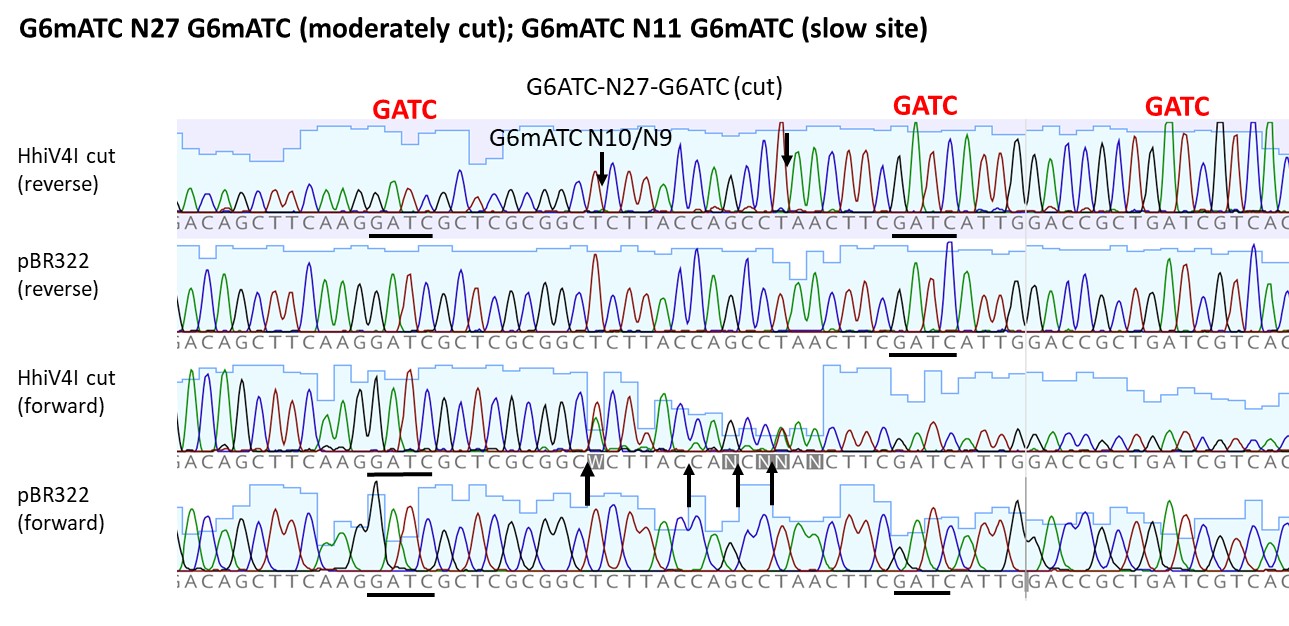


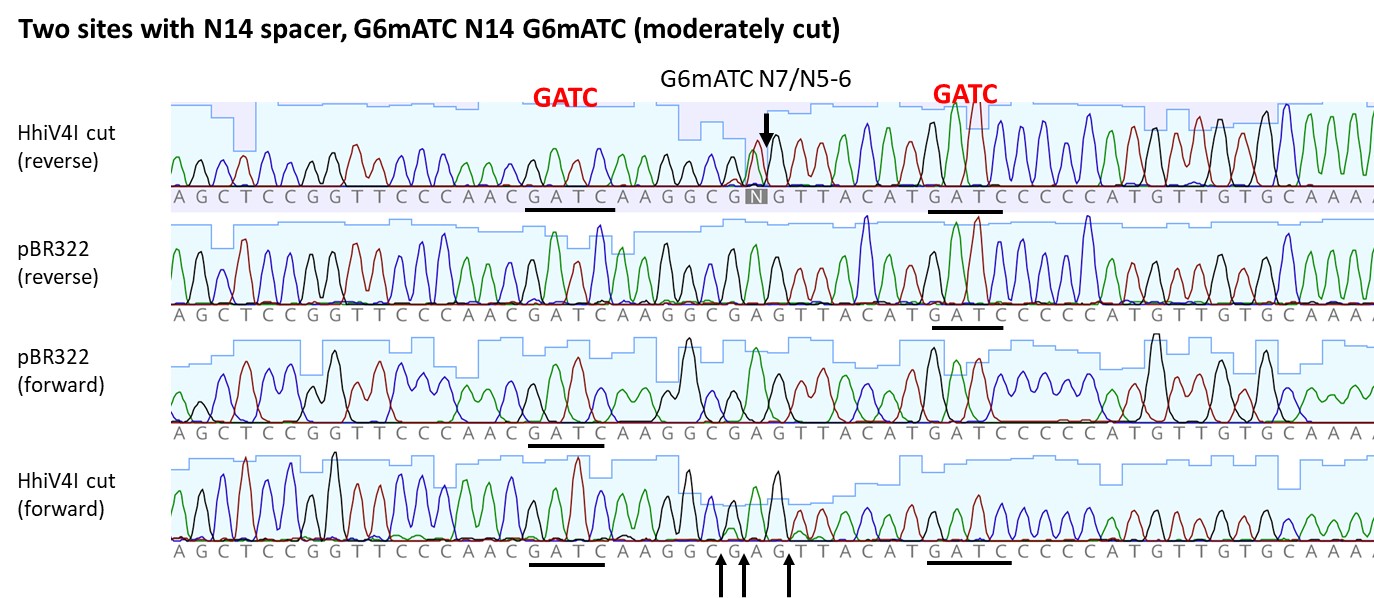


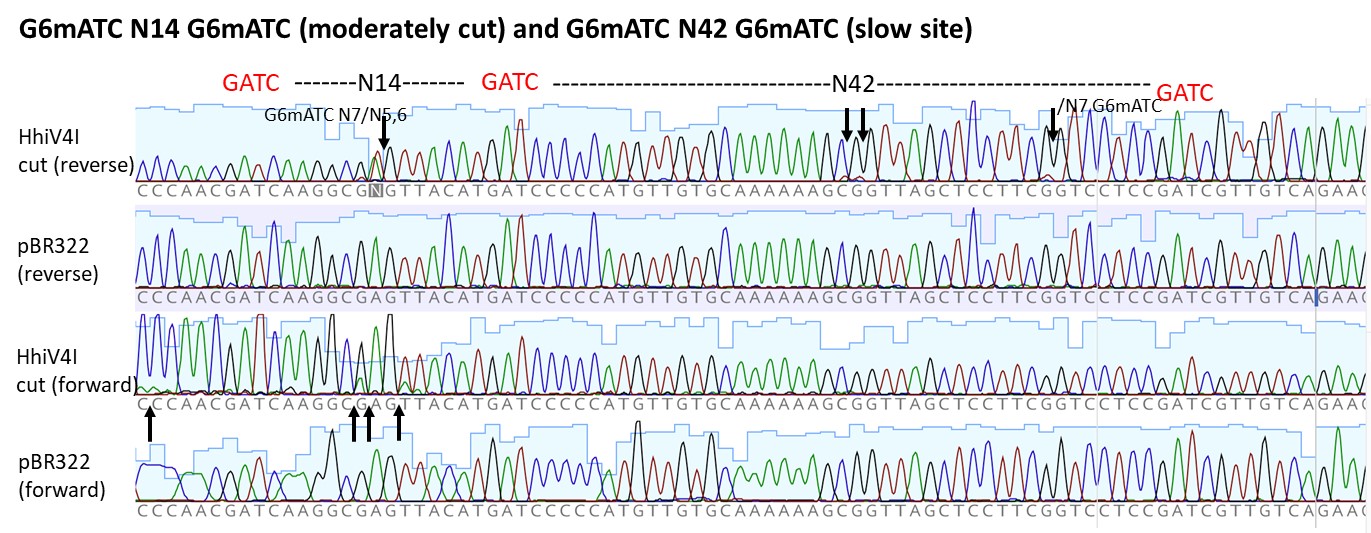


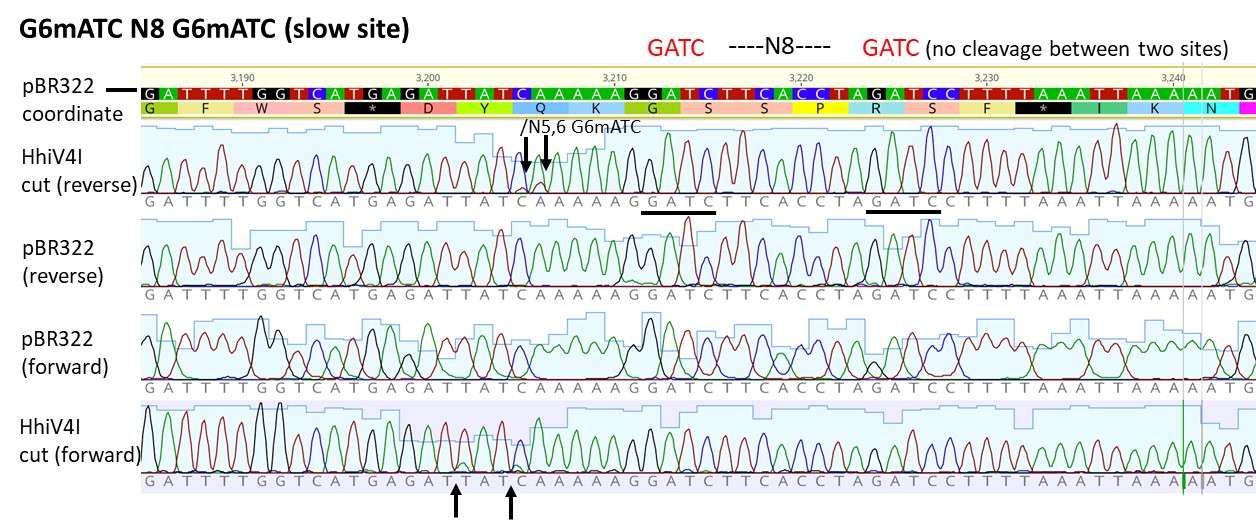


**Suppl. Fig. S7.** **DNA nicking activity of HtuIII endonuclease (PUA-wH—HNH fusion) and run-off sequencing of nicked pBR322**. **A** and **B**. Digestion of Dam^+^ pBR322 by HtuIII in Mn^2+^ and Co^2+^ buffer, respectively. **C**. run-off sequencing of digested pBR322 (Dam^+^). The nicking site is indicated by a down arrow (/N1 G6mATC N14 G6mATC), which occurred near two G6mATC sites in tandem. NC, nicked circular DNA; L, linear; SC, supercoiled DNA. HtuIII is active in Mn^2+^ and Co^2+^ buffers, and inactive in Mg^2+^ buffer (not shown), a property similar to HhiV4I.


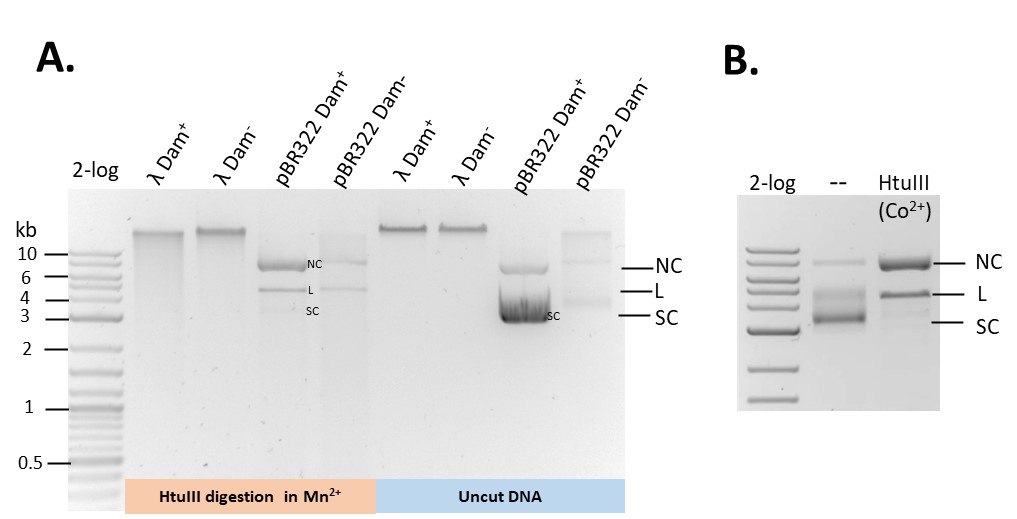


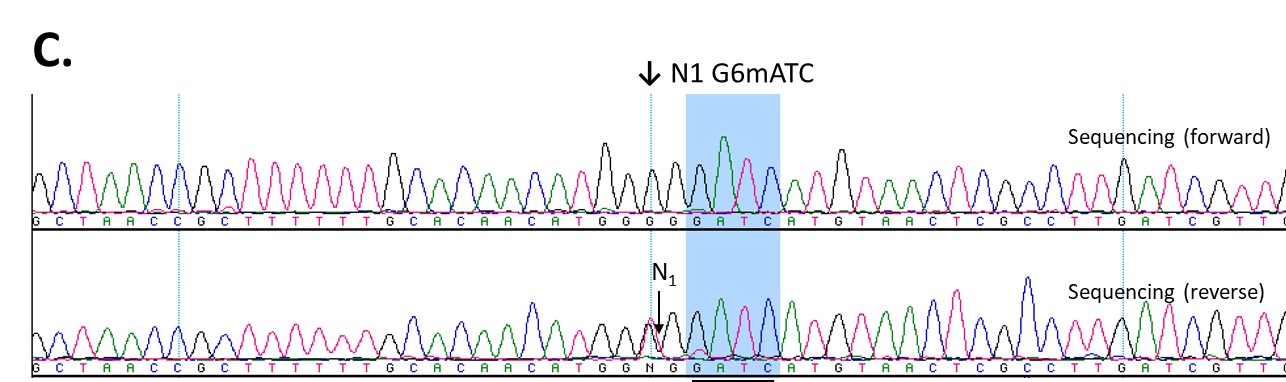


**Suppl. Fig. S8. Ahi29725I** **wH—GIY-YIG endonuclease activity in different divalent cations in digestion of Dam^+^ pBR322 (1 μg). A. Restriction activity assays in five different divalent cations in a medium salt buffer (50 mM NaCl, 20 mM Tris-HCl, pH 7.5).** Divalent cations were supplemented in a medium salt buffer at 10, 1, and 0.1 mM concentration in digests. DpnI and MboI were used as controls in CutSmart buffer. Ahi29725I generated discrete banding pattern in Mg^2+^ buffer. But it is also active in buffers with Mn^2+^, Co^2+^, and Ni^2+^, causing extensive smear. The smearing patterns may be caused by non-specific activity or star (relaxed) activity. The disappearing of DNA in Mn^2+^ and Co^2+^ buffers may result from digestion products of small fragments of less than 100 bp. In Ca^2+^ buffer (10 mM), the enzyme displayed strong nicking activity (conversion of supercoiled DNA to open circular form). Although majority of GIY-YIG endonucleases use Mg^2+^ as a cofactor for catalysis, the preferred divalent cation for Ahi29725I wH—GIY-YIG endonuclease in the native cells is still unknown. Addition of 10 mM EDTA completely inhibited Ahi29725I activity. SC, supercoiled DNA; OC (NC), open circular (nicked circular) DNA. **B.** Repeated restriction assay in Mg^2+^ or Mn^2+^ buffer with enzyme titration. Digested products were resolved in 2% agarose gel in order to detect small fragments. Note: large fragments of more than 3 kb and uncut substrates were not easily resolved in 2% agarose gel. Ahi (Ahi29725I endonuclease). Control digests: lane 1, Ahi digested in NEB buffer 2 (10 mM Mg^2+^); lane 2, DpnI digest pattern of Dam-modified pBR322. Negative control: DNA incubated with buffer and Ahi enzyme in the absence of divalent cations (no Me^2+^). Ahi is active in Mg^2+^ or Mn^2+^ buffer, but more smearing was detected in Mn^2+^ buffer, possibly as a result of non-specific cleavage. Enzyme titration: starting with 1 μg per reaction and 2-fold serial dilution of the enzyme in digestion of 1 μg of pBR322 (Dam^+^).

**A.**


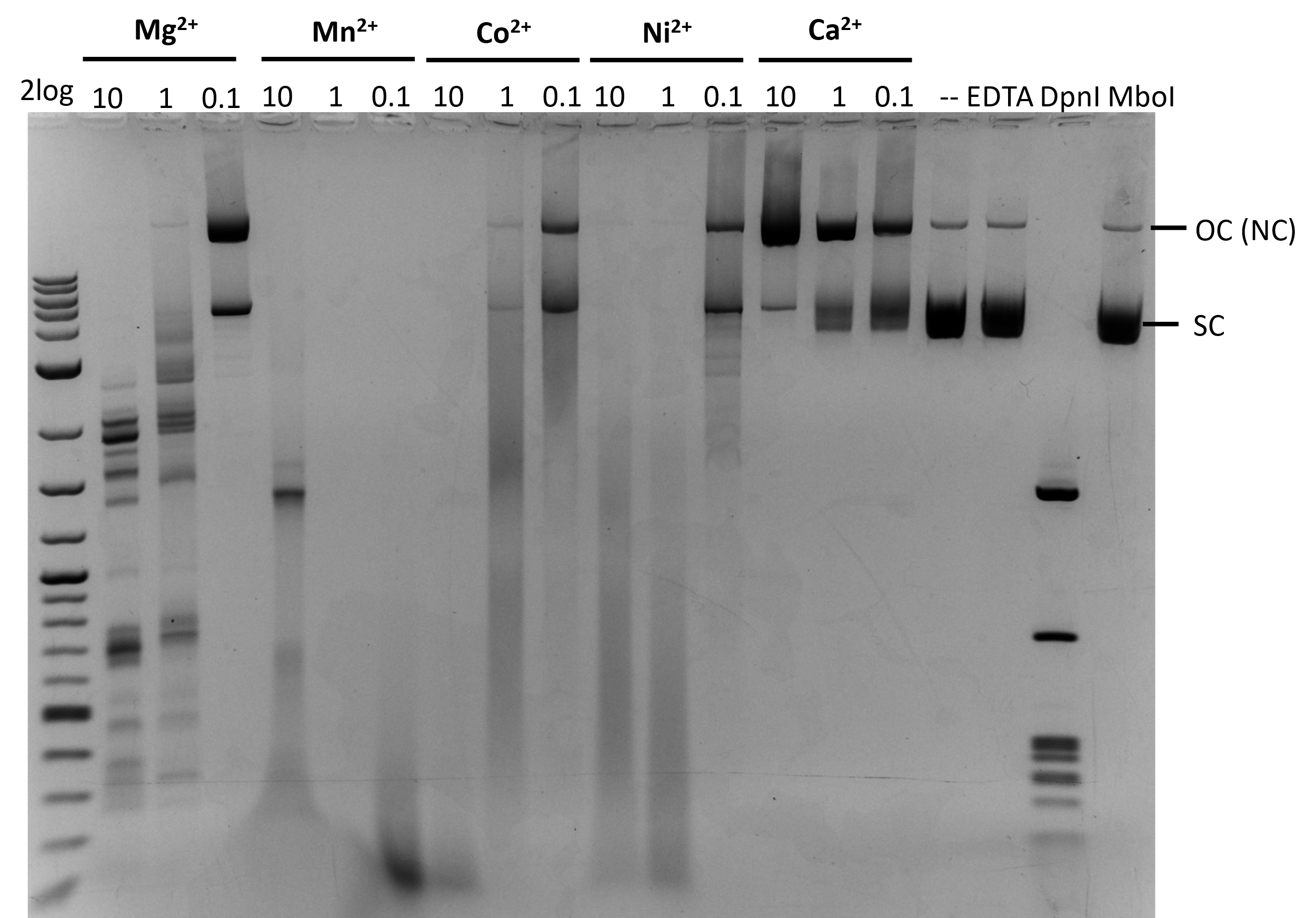


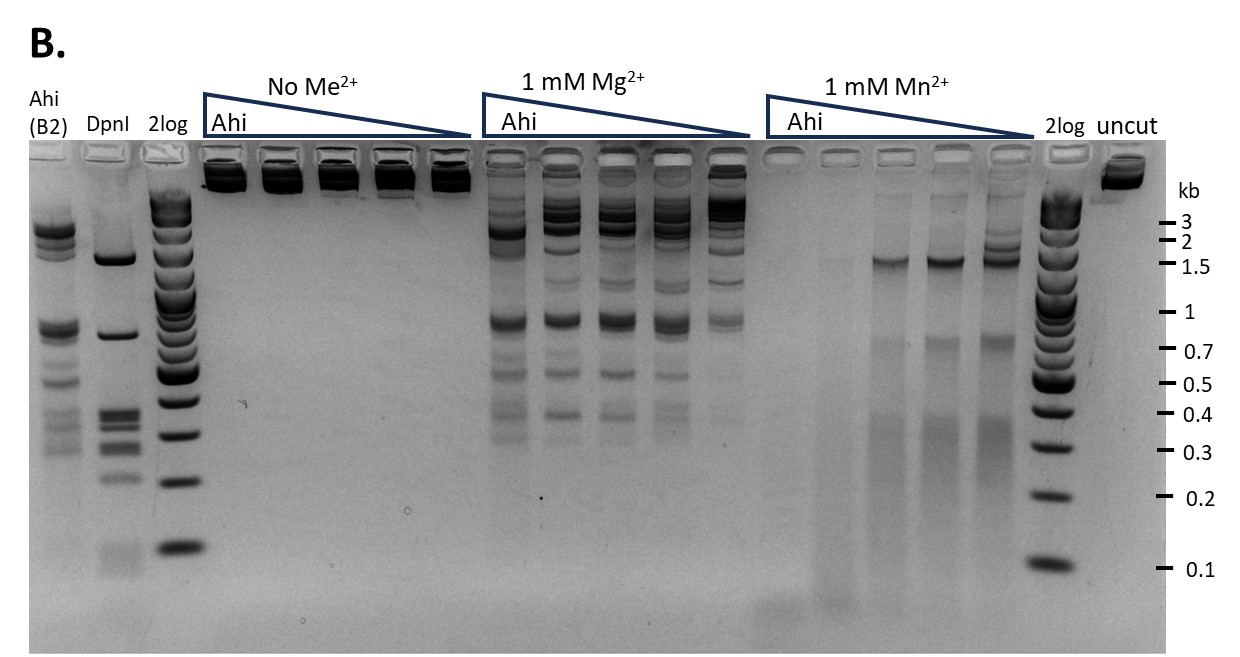


**Suppl. Fig. S9.** DNA run-off sequencing of Ahi29725I digested pBR322 (Dam^+^) to determine the cut sites outside of G6mATC at the cleavage distance of N1-23. The up and down arrows indicate the top and bottom strand cuts, respectively.


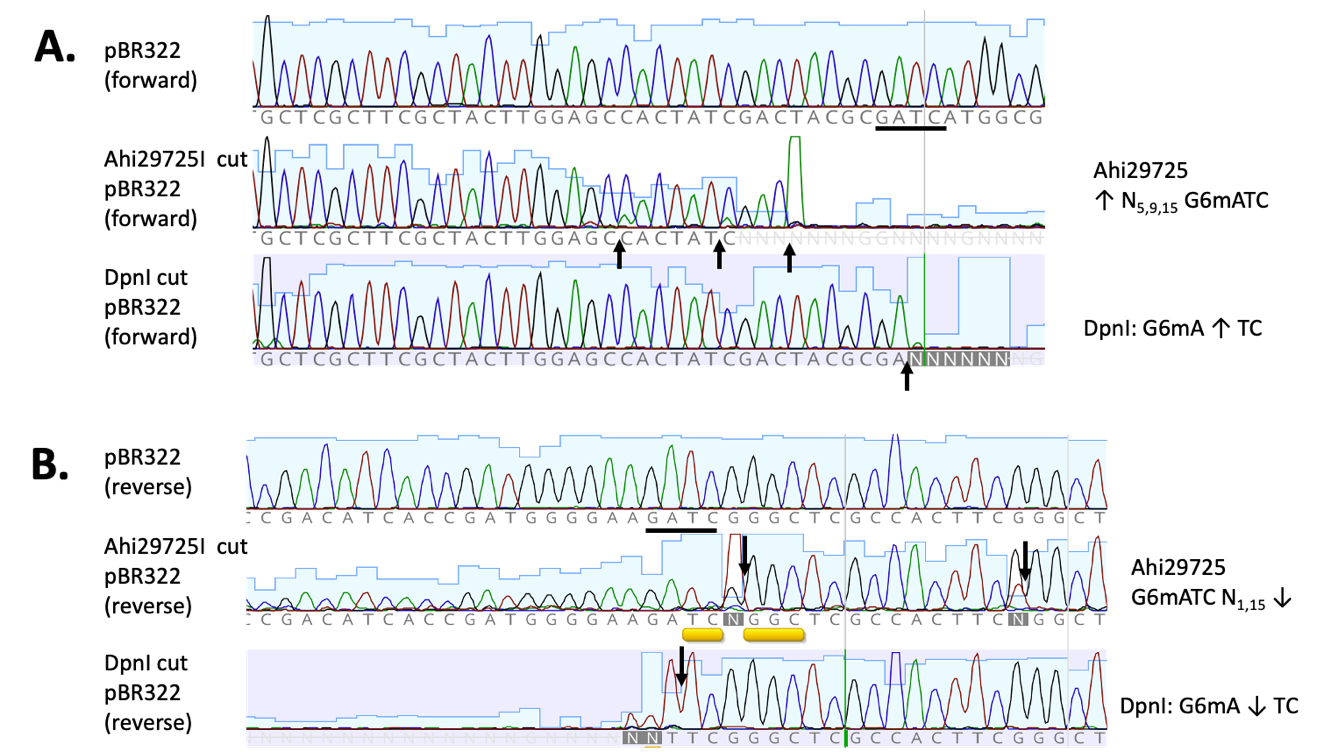


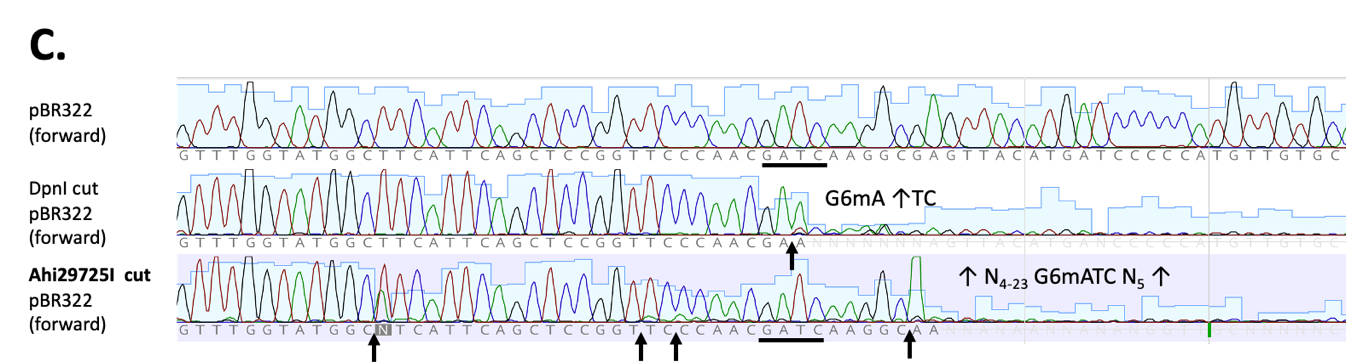


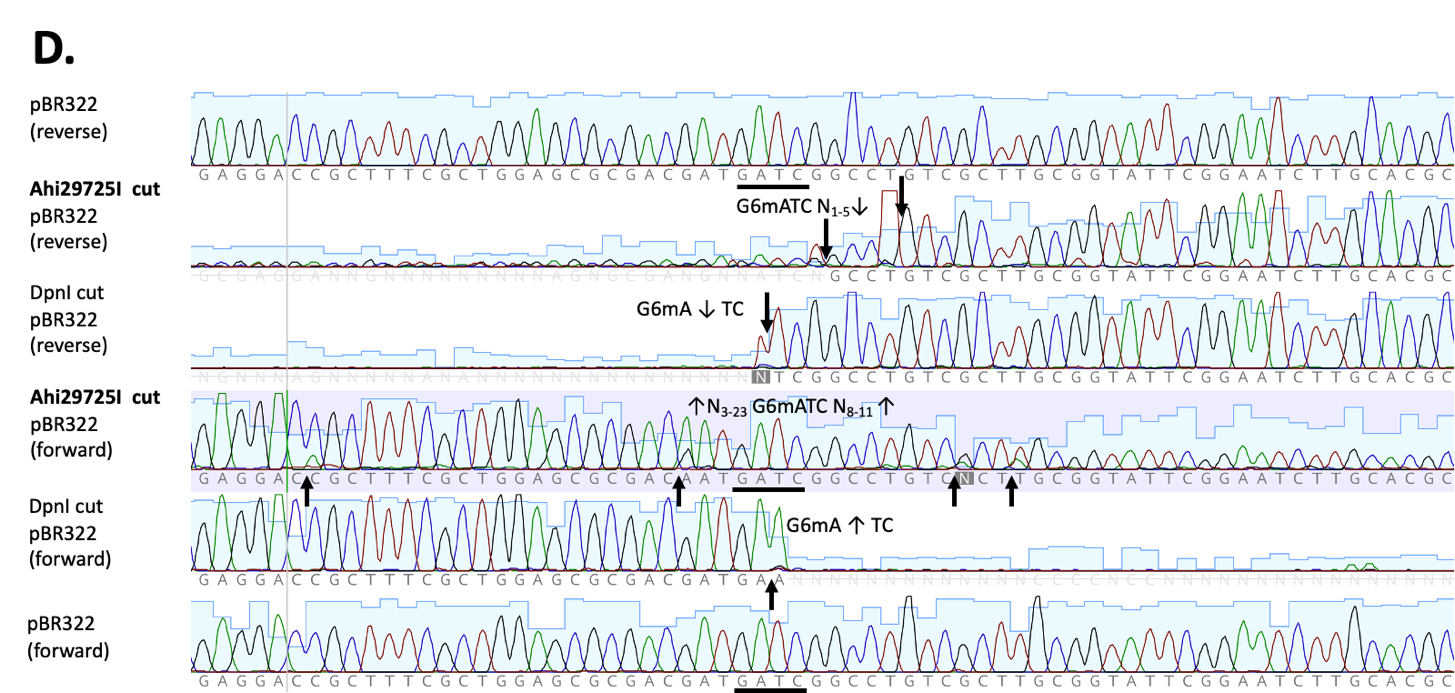


**Suppl. Fig. S10. DNA run-off sequencing to map Apa233I cleavage sites near modified G6mATC in digested pBR322 (Dam^+^).** The extra A or T peaks and dramatic decrease in peak height indicate the cleavage positions.


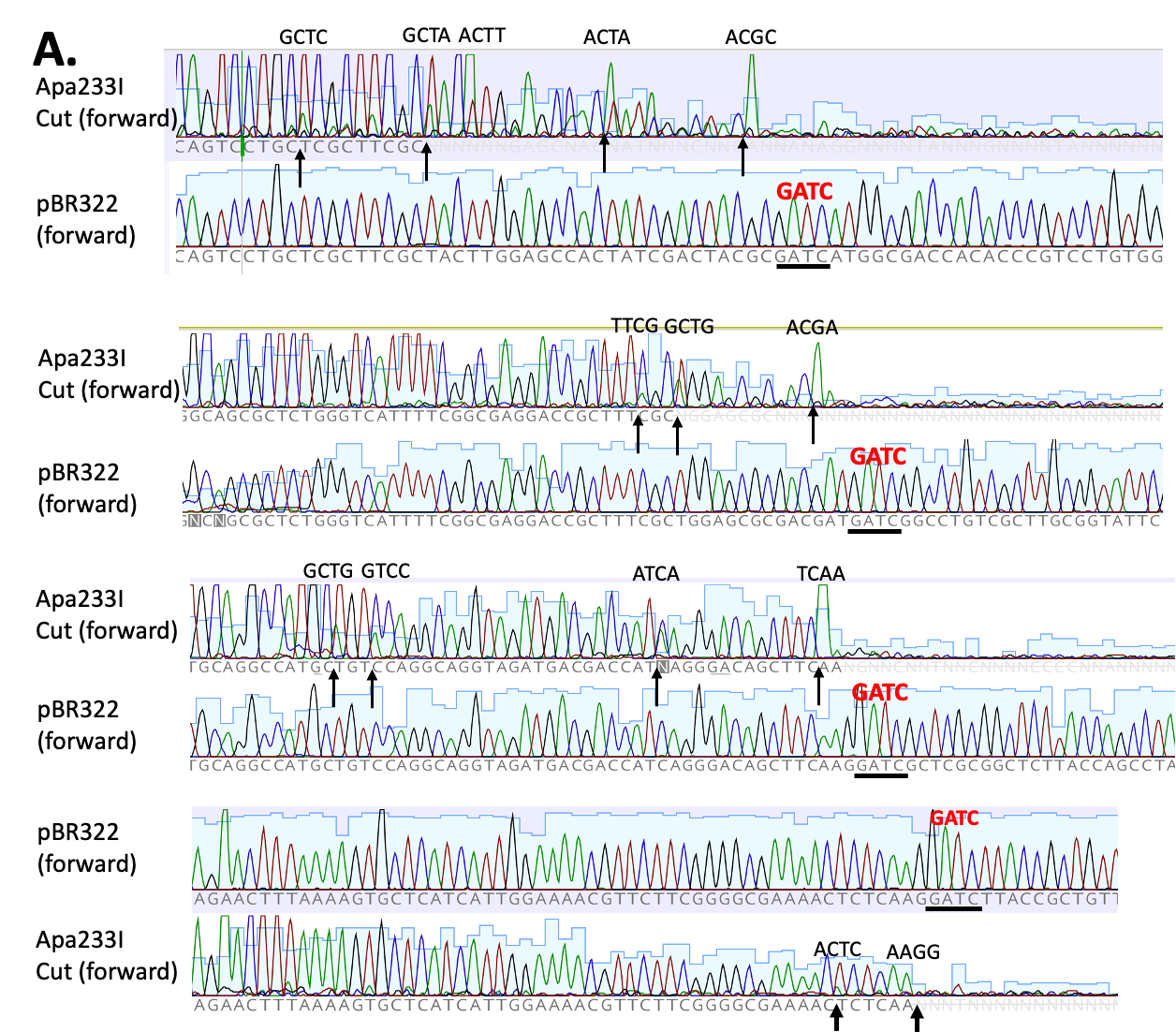


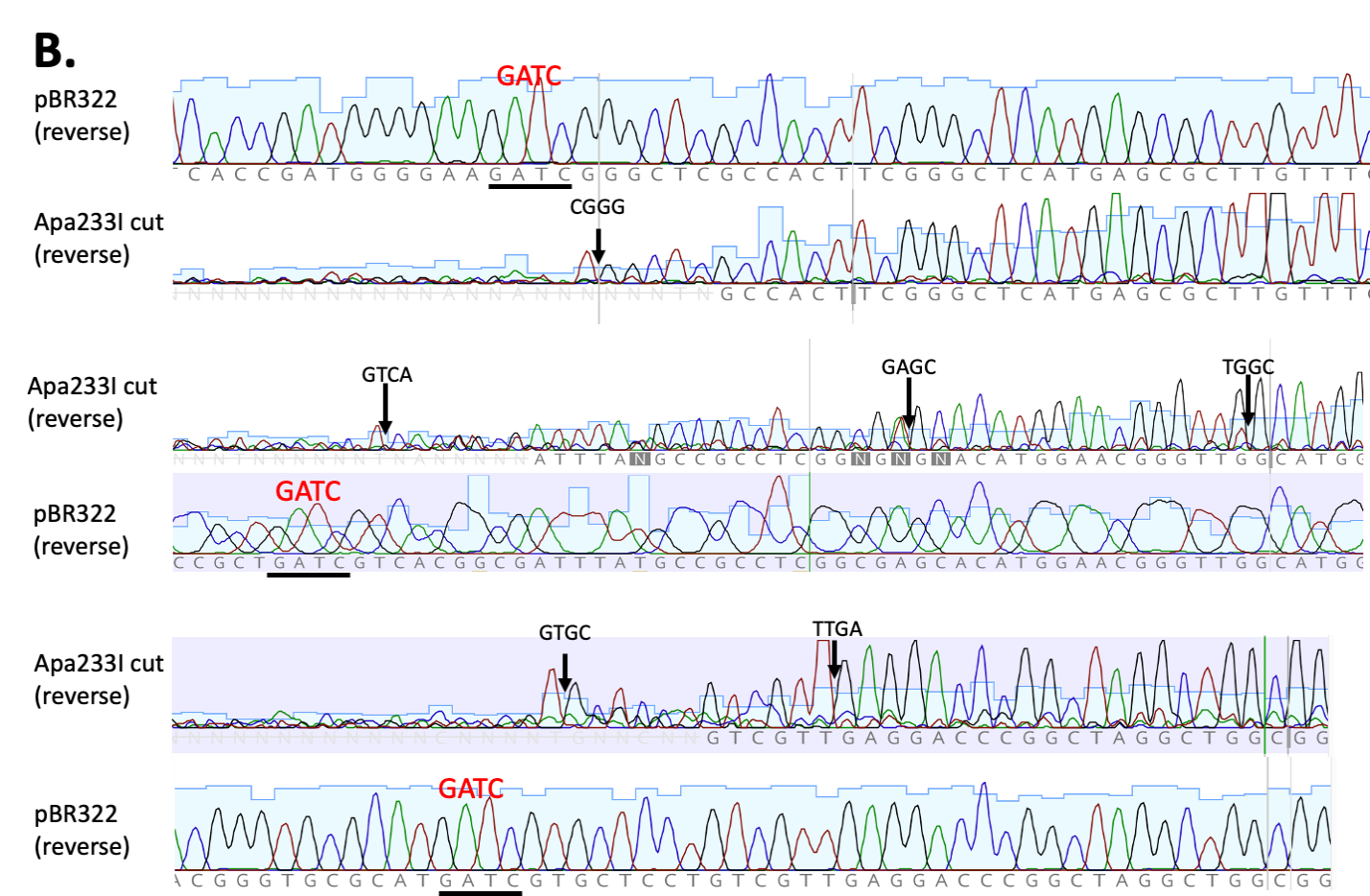


**Suppl. Fig. 11. The cut-site sequence preference of the GIY-YIG endonuclease domain of Ahi29725I (panels A, B) and Apa233I (panels C, D) as compiled by WebLogo** (https://weblogo.berkeley.edu/logo.cgi). For Ahi29725I, the top strand cleavages take place at 5′ NN/RN 3′ (complement strand cuts at 5′ NY/NN 3′). For Apa233I endonuclease, the cut site consensus sequence for the top strand cleavages appeared to be NN/GN. The bottom strand cut sites mostly occurred at NCNN or NTNN. The cleavage sites are located outside of the G6mATC sequences. The two GIY-YIG endonuclease domains have low sequence specificity compared to DpnI.


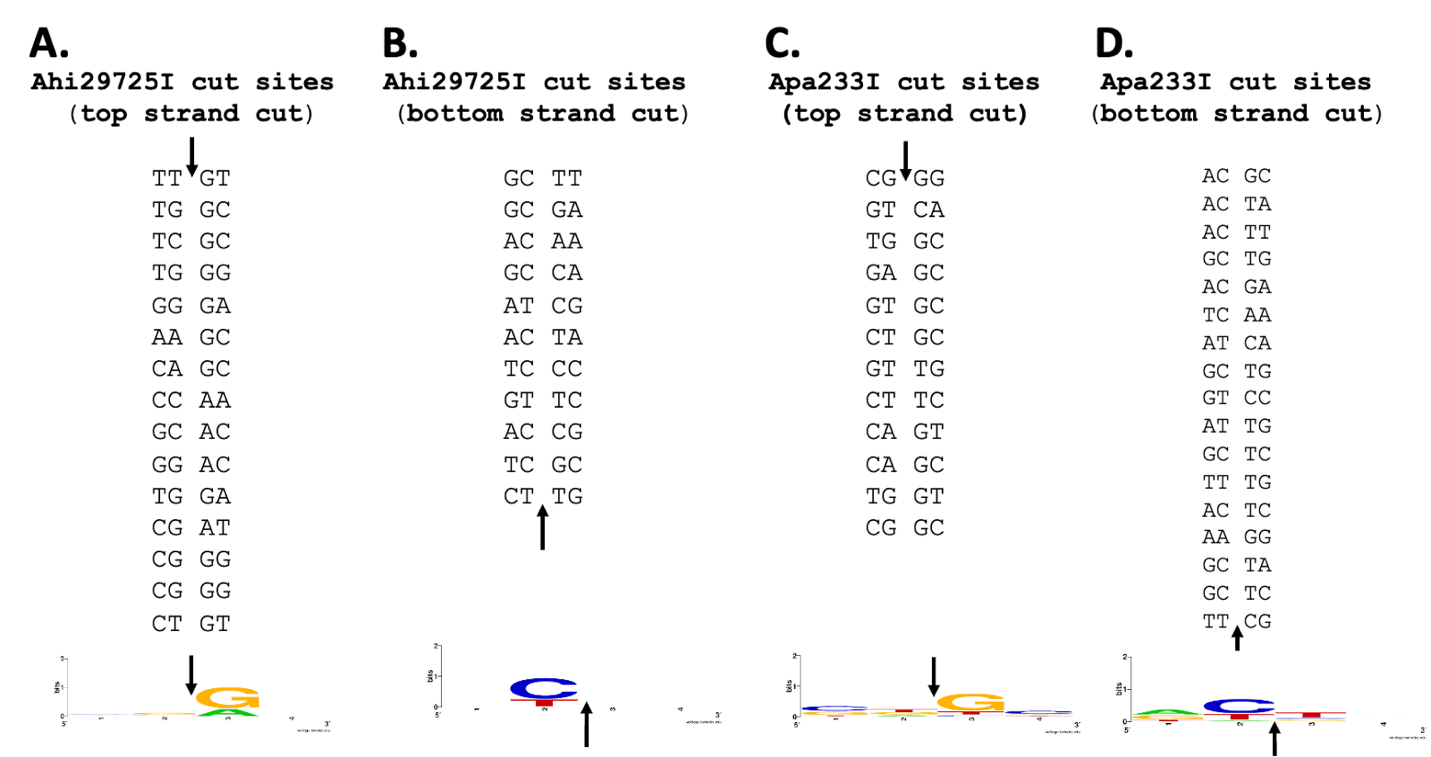


**Suppl. Fig. S12. Restriction digest of pBR322 (Dam^+^) and M.EcoGII-modified pBR322 (Dam^-^) with Ahi29725I (Ahi), Apa233I (Apa), DpnI, or MboI.** Three large fragments of Dam^+^ DNA (indicated by arrows) in a partial digestion by Ahi29725I (406.2 nM protein in 50 μl reaction volume) were further digested into small fragments after M.EcoGII methylation of Dam^-^ DNA. Apa233I (413.7 nM protein) was used to digest the substrates under the same digest condition. Modified G6mATC sites in pBR322 are resistant to MboI digestion and sensitive to DpnI. M.EcoGII, a frequent adenine methylase, is able to methylate most adenine bases except polyA tracks (2).


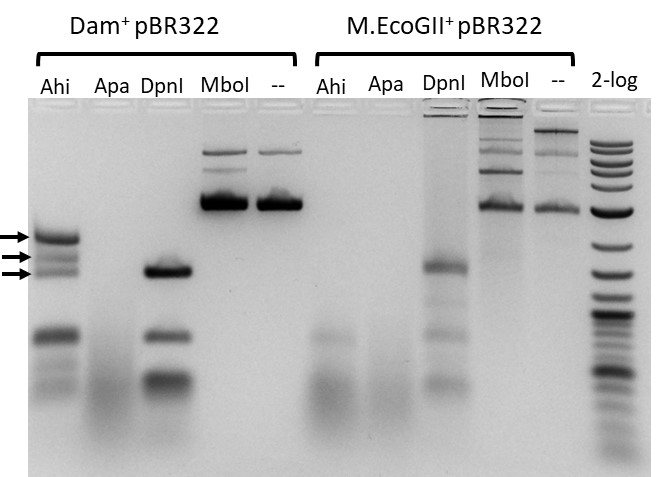


**References**

1. Xu, S.Y., Zhu, Z., Zhang, P. et al. Discovery of natural nicking endonucleases Nb.BsrDI and Nb.BtsI and engineering of top-strand nicking variants from BsrDI and BtsI (2007). *Nucleic* *Acids Res*, 35, 4608-18.
2. Murray, I.A., Morgan, R.D., Luyten, Y., Fomenkov, A., Correa, I.R., Jr., Dai, N., Allaw, M.B., Zhang, X., Cheng, X. and Roberts, R.J. (2018). The non-specific adenine DNA methyltransferase M.EcoGII. *Nucleic Acids Res*, 46, 840-848.

3. Weber, L.M., Jia, Y., Stielow, B., Gisselbrecht, S.S., Cao, Y., Ren, Y., Rohner, I., King, J., Rothman, E., Fischer, S. *et al.* (2023) The histone acetyltransferase KAT6A is recruited to unmethylated CpG islands via a DNA binding winged helix domain. *Nucleic Acids Res*, 51, 574-594.

**Raw data of modified and unmodified oligos digestions**

**Peakscan analysis of digested duplex oligos by Ahi29725I, Apa233I, HhiV4I, DpnI, FcyTI, and MboI, respectively.** Digestion of fully modified duplex oligos (M+/M+, panel **A**) by Ahi29725I, Apa233I, HhiV4I, DpnI, FcyTI, and MboI endonucleases, respectively. Panels **B** and **C**: digestion of hemi-modified duplex oligos (M+/M-, panel **B**; M-/M+, panel **C**) by the wH fusion endonucleases. Digestion of unmodified duplex oligos (M-/M-, panel **D**) by the fusion endonucleases.

Raw data: proteome analysis of the partially purified wH fusion endonucleases (see spreadsheet tables).

Suppl. Tables S4a,b (HhiV4I)

Suppl. Tables S5a,b (FcyTI)

Suppl. Tables S6a,b (Ahi29725I)

Suppl. Tables S7a,b (Apa233I)
